# Supplementary material for: Association Between Pre-Existing Conditions and COVID-19 Hospitalization, Intensive Care Services, and Mortality: A Cross-Sectional Analysis of an International Global Health Data Repository
Source: Pathogens. 2025 Sep 11;14(9):917. doi: 10.3390/pathogens14090917 (PMC12472582; doi:10.3390/pathogens14090917)
Supplement: Supplementary file 1 [file pathogens-14-00917-s001.zip › pathogens-3816492-supplementary/pathogens-3816492-supplementary-1.pdf]

Scheme 1. Countries reporting pre-existing conditions.

	pre-existing conditions						Outcome			Total
Country	CVD	HTN	Diabetes	Lung diseases	Kidney diseases	Obesity	Hosp	ICU	Mortality	
	Yes	Yes	Yes	Yes	Yes	Yes	Yes	Yes	Yes	
Brazil, n (%)	55,782 (3.0)	0	30,198 (1.6)	19,569 (1.0)	1,749 (0.1)	4092 (0.2)	6286 (0.3)	61 (0.0)	9016 (0.5)	1,880,335
Canada, n (%)	2 (0.0)	2 (0.0)	0	0	0	0	0	0	0	58,302
Cuba, n (%)	0 (0.00)	0	3 (0.0)	31 (0.1)	0	0	0	0	0	22,183
Gabon, n (%)	1 (16.7)	1 (16.7)	1 (16.7)	0	0	0	0	0	0	6
India, n (%)	1 (0.0)	1 (0.0)	1 (0.0)	0	0	0	2919 (14.4)	0	33 (0.2)	20,302
Mexico, n (%)	116,850 (13.1)	112,382 (12.6)	81,784 (9.2)	25,434 (2.9)	9,222 (1.0)	91,230 (10.2)	77,718 (8.7)	4083 (0.5)	18,597 (2.1)	893,738
Moldova, n (%)	1 (0.0)	1 (0.0)	1 (0.0)	0	0	1 (0.0)	0	0	0	11,067
Nigeria, n (%)	0	0	1 (0.0)	0	0	0	0	0	0	6403
South Korea, n (%)	0	0	0	2 (0.0)	0	0	0	9 (0.1)	36 (0.3)	14,544
Vietnam, n (%)	1 (3.1)	1 (3.1)	1 (3.1)	0	0	0	0	26 (81.3)	0	32

“No” may contain missing

All percentages are rows

Abbreviations: CVD (cardiovascular disease including hypertension), HTN (hypertension), ICU (intensive care services)

Supplementary Table 2 - Characteristics and outcomes of cases from countries reporting pre-existing conditions (Brazil, Cuba, and Mexico)

	Overall	Hospitalization			ICU			Mortality		
		Yes, N = 83,183 (4.3%)	No, N = 1 836,107 (95.7%)	P-value	Yes, N= 4 130 (0.2%)	No, N= 1 915,160 (99.8%)	P- value	Yes, N= 27,072 (1.4%)	No, N= 1 892,218 (98.6%)	P- value

Gender n (%)									
Female	1,009,988 (52.6)	36,469 (3.6)	973,519 (96.4)		1653 (0.2)	1,008,335 (99.8)		10,558 (1.1)	999,430 (99.0)	
Male	909,302 (47.4)	46,714 (5.1)	862,588 (94.9)	<0.01	2477 (0.3)	906,825 (99.7)	<0.01	16,514 (1.8)	892,788 (98.2)	<0.01
Missing	338 (0.0)									
Age groups, n (%)										
0-9 yrs	63,613 (3.3)	4350 (6.8)	59,263 (93.2)		252 (0.4)	63,361 (99.6)		158 (0.3)	63,455 (99.8)	
10-19 yrs	108,642 (5.7)	1957 (1.8)	106,685 (98.2)		76 (0.1)	108,566 (99.9)		83 (0.1)	108,559 (99.9)	
20-29 yrs	372,832 (19.4)	4279 (1.2)	368,553 (98.9)		165 (0.0)	372,667 (100.0)		375 (0.1)	372,457 (99.9)	
30-39 yrs	439,122 (22.9)	6930 (1.6)	432,192 (98.4)		300 (0.1)	438,822 (99.9)		1038 (0.2)	438,084 (99.8)	
40-49 yrs	379,180 (19.8)	11,064 (2.9)	368,116 (97.1)		505 (0.1)	378,675 (99.9)		2626 (0.7)	376,554 (99.3)	
50-59 yrs	280,179 (14.6)	15,707 (5.6)	264,472 (94.4)		807 (0.3)	279,372 (99.7)		4831 (1.7)	275,348 (98.3)	
60-69 yrs	150,070 (7.8)	17,863 (11.9)	132,207 (88.1)		941 (0.6)	149,129 (99.4)		6492 (4.3)	143,578 (95.7)	
70-79 yrs	74,989 (3.9)	13,611 (18.2)	61,378 (81.9)		725 (1.0)	74,264 (99.0)		6135 (8.2)	68,854 (91.8)	
80-89 yrs	26,762 (1.4)	6445 (24.1)	20,317 (75.9)		305 (1.1)	26,457 (98.9)		3236 (12.1)	23,526 (87.9)	
90-99 yrs	3971 (0.2)	923 (23.2)	3048 (76.8)		47 (1.2)	3924 (98.8)		491 (12.4)	3480 (87.6)	
100-109 yrs	350 (0.0)	36 (10.3)	314 (89.7)		5 (1.4)	345 (98.6)		25 (7.1)	325 (92.9)	
110-120 yrs	192 (0.0)	18 (9.4)	174 (90.6)	<0.01	2 (1.0)	190 (99.0)	<0.01	14 (7.3)	178 (92.7)	<0.01
Country										
Brazil, n (%)	1,015,975 (52.9)	5729 (0.6)	1,010,246 (99.4)		61 (0.0)	1,015,914 (100.0)		5569 (0.8)	1,007,406 (99.2)	

Mexico, n (%)	893,167 (46.5)	77,454 (8.7)	815,713 (91.3)		4069 (0.5)	889,098 (99.5)		18,503 (2.1)	874,664 (97.9)	
Cuba, n (%)	10,486 (0.5)	0	10,486 (100.0)	<0.01	0	10,486 (100.0)	<0.01	0	10,486 (100.0)	<0.01
Year of diagnosis										
2020, n (%)	1,070,514 (55.8)	26,892 (2.5)	1,043,622 (97.5)		1666 (0.2)	1,068,848 (99.8)		15,301 (1.4)	1,055,213 (98.6)	
2021, n (%)	849,114 (44.2)	56,291 (6.6)	792,823 (93.4)	<0.01	2464 (0.3)	846,650 (99.7)	<0.01	11,771 (1.4)	837,343 (98.6)	0.01
Period of diagnosis										
Jan-Mar 2020	29,805 (1.6)	6232 (20.9)	23,573 (79.1)		86 (0.3)	29,719 (99.7)		838 (2.8)	28,967 (97.2)	
Apr-Jun 2020	336,763 (17.5)	5327 (1.6)	331,436 (98.4)		326 (0.1)	336,437 (99.9)		5894 (1.8)	330,869 (98.3)	
Jul- Sep 2020	371,038 (19.3)	5927 (1.6)	365,111 (98.4)		400 (0.1)	370,638 (99.9)		3958 (1.1)	367,080 (98.9)	
Oct-Dec 2020	332 908 (17.3)	9406 (2.8)	323,502 (97.2)		854 (0.3)	332,054 (99.7)		4611 (1.4)	328,297 (98.6)	
Jan-Mar 2021	849 114 (44.2)	56,291 (6.6)	792,823 (93.4)	<0.01	2464 (0.3)	846,650 (99.7)	<0.01	11,771 (1.4)	837,343 (98.6)	<0.01

Supplementary Table 3 - Proportion of cases with preexisting conditions hospitalized by age group

Hospitalizations by age groups	Cardiovascular Disease			Diabetes			Lung Disease			Kidney Disease			Hypertension			Obesity		
	Yes, n (%)	No, n (%)	P value	Yes, n (%)	No, n (%	P value	Yes, n (%)	No, n (%)	P value	Yes, n (%)	No, n (%)	P value	Yes, n (%)	No, n (%)	P value	Yes, n (%)	No, n (%)	P value
Overall, hospitalized	30,403 (17.6)	53,601 (2.0)		24,019 (21.5)	59,985 (2.2)		5090 (11.3)	78,914 (2.9)		4915 (44.8)	79,089 (2.8)		26,836 (23.9)	57 168 (2.1)		13,301 (14.0)	70,703 (2.6)	

Overall, not hospitalized,	142,229 (82.4)	2 570 023 (98.0)	<0.01	87，966 (78.6)	2,624,286 (97.8)	<0.01	39,944 (88.7)	2672 308 (97.1)	<0.01	6056 (55.2)	2,706,196 (97.2)	<0.01	85,546 (76.1)	2,626,706 (97.9)	<0.01	82 021 (86.1)	2 630 231 (97.4)	<0.01
0-9 yrs, hospitalized	173 (33.1)	4177 (6.6)	<0.01	26 (15.6)	4324 (6.8)	<0.01	190 (13.1)	4160 (6.7)	<0.01	19 (24.1)	4331 (6.8)	<0.01	23 (28.8)	4327 (6.8)	<0.01	38 (14.1)	4 312 (6.8)	<0.01
0-9 yrs, not hospitalized	350 (66.9)	58，913 (93.4)		141 (84.4)	59,122 (93.2)		1256 (86.9)	58,007 (93.3)		60 (76.0)	59,203 (93.2)		57 (71.3)	59,206 (93.2)		231 (85.9)	59,032 (93.2)	
10-19 yrs, hospitalized	66 (10.7)	1891 (1.8)	<0.01	67 (15.0	1890 (1.8)	<0.01	114 (3.9)	1843 (1.7)	<0.01	71 (39.2)	1,886 (1.7)	<0.01	38 (16.0)	1919 (1.8)	<0.01	104 (5.3)	1853 (1.7)	<0.01
10-19 yrs, not hospitalized	549 (89.3)	106，136 (98.3)		379 (85.0)	106,306 (98.3)		2823 (96.1)	103,862 (98.3)		110 (60.8)	106,575 (98.3)		199 (84.0)	106,486 (98.2)		1853 (94.7)	104,832 (98.3)	
20-29 yrs, hospitalized	385 (8.22)	3894 (1.1)	<0.01	240 (11.0)	4039 (1.1)	<0.01	155 (2.0)	4124 (1.1)	<0.01	269 (30.9)	4010 (1.1)	<0.01	344 (11.7)	3935 (1.1)	<0.01	481 (3.4)	3798 (1.1)	<0.01
20-29 yrs, not hospitalized	4299 (91.8)	364,254 (98.9)		1939 (89.0)	366,614 (98.9)		7748 (98.0)	360,805 (98.9)		603 (69.2)	367,950 (98.9)		2591 (88.3)	365,962 (98.9)		13,549 (96.6)	355,004 (98.9)	
30-39 yrs, hospitalized	884 (6.6)	6046 (1.4)	<0.01	695 (9.5)	6235 (1.4)	<0.01	241 (3.0)	6689 (1.6)	<0.01	402 (32.4)	6528 (1.5)	<0.01	773 (9.4)	6157 (1.4)	<0.01	1252 (5.7)	5678 (1.4)	<0.01
30-39 yrs, not hospitalized	12,434 (93.4)	419,758 (98.6)		6635 (90.5)	425,557 (98.6)		7847 (97.0)	424,345 (98.5)		838 (67.6)	431,354 (98.5)		7468 (90.6)	424,724 (98.6)		20,800 (94.3)	411,392 (98.6)	
40-49 yrs, hospitalized	2585 (8.7)	8479 (2.4)	<0.01	2543 (13.1)	8521 (2.4)	<0.01	413 (5.5)	10,651 (2.9)	<0.01	550 (35.7)	10,514 (2.8)	<0.01	2315 (11.7)	8749 (2.4)	<0.01	2335 (10.2)	8729 (2.5)	<0.01
40-49 yrs, not hospitalized	27,250 (91.3)	340,866 (97.6)		16,820 (86.9)	351,296 (97.6)		7086 (94.5)	361,030 (97.1)		990 (64.3)	367 126 (97.2)		17 454 (88.3)	350,662 (97.6)		20,616 (89.8)	347,500 (97.6)	
50-59 yrs, hospitalized	5747 (12.9)	9960 (4.2)	<0.01	5284 (17.0)	10,423 (4.2)	<0.01	679 (11.2)	15,028 (5.5)	<0.01	1000 (44.4)	14,707 (5.3)	<0.01	5176 (17.2)	10,531 (4.2)	<0.01	3130 (17.1)	12,577 (4.8)	<0.01
50-59 yrs, not hospitalized	38,723 (87.1)	225,749 (95.8)		25,827 (83.0)	238,645 (95.8)		5410 (88.9)	259,062 (94.5)		1250 (55.6)	263,222 (94.7)		24,972 (82.8)	239,500 (95.8)		15,187 (82.9)	249,285 (95.2)	
60-69 yrs, hospitalized	8524 (22.0)	9339 (8.4)	<0.01	7207 (25.4)	10,656 (8.8)	<0.01	1084 (23.8)	16,779 (11.5)	<0.01	1308 (56.3)	16,555 (11.2)	<0.01	7633 (28.6)	10,230 (8.3)	<0.01	3176 (32.4)	14,687 (10.5)	<0.01

60-69 yrs, not hospitalized	30,307 (78.1)	101,900 (91.6)		21,148 (74.6)	111,059 (91.3)		3463 (76.2)	128,744 (88.5)		1013 (43.6)	131,194 (88.8)		19,017 (71.4)	113,190 (91.7)		6615 (67.6)	125,592 (89.5)	
70-79 yrs, hospitalized	7420 (30.3)	6191 (12.3)	<0.01	5383 (33.1)	8228 (14.0)	<0.01	1176 (34.5)	12,435 (17.4)	<0.01	843 (58.8)	12,768 (17.4)	<0.01	6564 (40.1)	7047 (12.0)	<0.01	1988 (46.9)	11,623 (16.4)	<0.01
70-79 yrs, not hospitalized	17,058 (69.7)	44,320 (87.7)		10,875 (66.9)	50,503 (86.0)		2229 (65.5)	59,149 (82.6)		590 (41.2)	60,788 (82.6)		9810 (59.9)	51,568 (88.0)		2252 (53.1)	59,126 (83.6)	
80-89 yrs, hospitalized	3676 (38.2)	2769 (16.2)	<0.01	2151 (40.6)	4294 (20.0)	<0.01	785 (46.0)	5660 (22.6)	<0.01	369 (59.0)	6076 (23.3)	<0.01	3363 (49.6)	3082 (15.4)	<0.01	689 (53.0)	5756 (22.6)	<0.01
80-89 yrs, not hospitalized	5937 (61.8)	14,380 (83.9)		3151 (59.4)	17,166 (80.0)		922 (54.0)	19 395 (77.4)		256 (41.0)	20,061 (76.8)		3420 (50.4)	16,897 (84.6)		610 (47.0)	19,707 (77.4)	
90-99 yrs, hospitalized	483 (40.8)	440 (15.8)	<0.01	201 (42.1)	722 (20.7)	<0.01	122 (44.0)	801 (21.7)	<0.01	37 (53.6)	886 (22.7)	<0.01	446 (53.4)	477 (15.2)	<0.01	61 (55.0)	862 (22.3)	<0.01
90-99 yrs, not hospitalized	702 (59.2)	2346 (84.2)		277 (58.0)	2771 (79.3)		155 (56.0)	1893 (78.3)		32 (46.4)	3016 (77.3)		390 (46.7)	2658 (84.8)		50 (45.1)	2998 (77.7)	
100-109 yrs, hospitalized	15 (25.4)	21 (7.2)	<0.01	6 (26.1)	30 (9.2)	0.02	6 (31.6)	30 (9.1)	0.01	4 (100.0)	32 (9.3)	<0.01	13 (43.3)	23 (7.2)	<0.01	2 (33.3)	34 (9.9)	0.12
100-109 yrs, not hospitalized	44 (74.6)	270 (92.8)		17 (73.9)	297 (90.8)		13 (68.4)	301 (90.9)		0 (0.0)	314 (90.8)		17 (56.7)	297 (92.8)		4 (66.7)	310 (90.1)	
110-120 yrs, hospitalized	6 (22.2)	12 (7.3)	0.03	5 (38.5)	13 (7.3)	<0.01	3 (30.0)	15 (8.2)	0.06	NA	18	NA	6 (46.2)	12 (6.7)	<0.01	1 (20.0)	17 (9.1)	0.39
110-120 yrs, not hospitalized	21 (77.8)	153 (92.7)		8 (61.5)	166 (92.7)		7 (70.0)	167 (91.8)		NA	174		7 (53.9)	167 (93.3)		4 (80.0)	170 (90.9)	

Abbreviations: CVD (cardiovascular disease including hypertension), HTN (hypertension), ICU (intensive care unit)

Supplementary Table 4 - Proportion of cases with preexisting conditions in ICU by age group

ICU by age groups	Cardiovascular disease			Diabetes			Lung Disease			Kidney Disease			Hypertension			Obesity		
	Yes, n (%)	No, n (%)	*p* value	Yes, n (%)	No, n (%)	*p* value	Yes, n (%)	No, n (%)	*p* value	Yes, n (%)	No, n (%)	*p* value	Yes, n (%)	No, n (%)	*p* value	Yes, n (%)	No, n (%)	*p* value

Overall, ICU	1567 (0.9)	2577 (0.1)		1236 (1.1)	2908 (0.1)		194 (0.4)	3950 (0.1)		168 (1.5)	3976 (0.1)		1472 (1.3)	2672 (0.1)		928 (1.0)	3216 (0.1)	
Overall, No ICU	171,065 (99.1)	2621047 (99.9)	<0.01	110,749 (98.9)	2,681,363 (99.9)	<0.01	44,840 (99.6)	2,747,272 (99.9)	<0.01	10,803 (98.5)	2,781,309 (99.9)	<0.01	110,910 (98.7)	2,681,202 (99.9)	<0.01	94,394 (99.0)	2,697,718 (99.9)	<0.01
0-9 yrs ICU	10 (1.9)	242 (0.4)	<0.01	1 (0.6)	251 (0.4)	0.49	2 (0.1)	250 (0.4)	0.11	1 (1.3)	251 (0.4)	0.27	1 (1.3)	251 (0.4)	0.27	0	252 (0.4)	0.63
0-9 yrs, No ICU	513 (98.1)	62,848 (99.6)		166 (99.4)	63,195 (99.6)		1444 (99.9)	61,917 (99.6)		78 (98.7)	63,283 (99.6)		79 (98.8)	63,282 (99.6)		269 (100.0)	63,092 (99.6)	
10-19 yrs, ICU	3 (0.5)	73 (0.1)	0.01	3 (0.7)	73 (0.1)	<0.01	2 (0.1)	74 (0.1)	1.00	1 (0.6)	75 (0.1)	0.12	1 (0.4)	75 (0.1)	0.15	10 (0.5)	66 (0.1)	<0.01
10-19 yrs, No ICU	612 (99.5)	107,954 (99.9)		443 (99.3)	108,123 (99.9)		2935 (99.9)	105,631 (99.9)		180 (99.5)	108,386 (99.9)		236 (99.6)	108,330 (99.9)		1947 (99.5)	106,619 (99.9)	
20-29 yrs, ICU	12 (0.3)	153 (0.0)	<0.01	18 (0.8)	147 (0.0)	<0.01	6 (0.1)	159 (0.0)	0.17	7 (0.8)	158 (0.0)	<0.01	10 (0.3)	155 (0.0)	<0.01	32 (0.2)	133 (0.0)	<0.01
20-29 yrs, No ICU	4672 (99.7)	367,996 (100.0)		2161 (99.2)	370,506 (100.0)		7897 (99.9)	364,770 (100.0)		865 (99.2)	371,802 (100.0)		2925 (99.7)	369,742 (100.0)		13,998 (99.8)	358,669 (100.0)	
30-39 yrs, ICU	43 (0.3)	257 (0.1)	<0.01	41 (0.6)	259 (0.1)	<0.01	9 (0.1)	291 (0.1)	0.14	16 (1.3)	284 (0.1)	<0.01	40 (0.5)	260 (0.1)	<0.01	86 (0.4)	214 (0.1)	<0.01
30-39 yrs, No ICU	13,275 (99.7)	425,547 (99.9)		7289 (99.4)	431,533 (99.9)		8079 (99.9)	430,743 (99.9)		1224 (98.7)	437,598 (99.9)		8201 (99.5)	430,621 (99.9)		21,966 (99.6)	416,856 (100.0)	
40-49 yrs, ICU	110 (0.4)	395 (0.1)	<0.01	122 (0.6)	383 (0.1)	<0.01	22 (0.3)	483 (0.1)	<0.01	19 (1.2)	486 (0.1)	<0.01	101 (0.5)	404 (0.1)	<0.01	162 (0.7)	343 (0.1)	<0.01
40-49 yrs, No ICU	29,725 (99.6)	348,950 (99.9)		19,241 (99.4)	359,434 (99.9)		7477 (99.7)	371,198 (99.9)		1521 (98.8)	377,154 (99.9)		19,688 (99.5)	359,007 (99.9)		22,789 (99.3)	355,886 (99.9)	
50-59 yrs, ICU	284 (0.6)	523 (0.2)	<0.01	289 (0.9)	518 (0.2)	<0.01	30 (0.5)	777 (0.3)	<0.01	31 (1.4)	776 (0.3)	<0.01	273 (0.9)	534 (0.2)	<0.01	207 (1.1)	600 (0.2)	<0.01
50-59 yrs, No ICU	44,186 (99.4)	235,186 (99.8)		30,822 (99.1)	248,550 (99.8)		6059 (99.5)	273,313 (99.7)		2219 (98.6)	277,153 (99.7)		29,875 (99.1)	249,497 (99.8)		18,110 (98.9)	261,262 (99.8)	
60-69 yrs, ICU	469 (1.2)	472 (0.4)	<0.01	379 (1.3)	562 (0.5)	<0.01	45 (1.0)	896 (0.6)	<0.01	44 (1.9)	897 (0.6)	<0.01	443 (1.7)	498 (0.4)	<0.01	232 (2.4)	709 (0.5)	<0.01

60-69 yrs, No ICU	38,362 (98.8)	110,767 (99.6)		27,976 (98.7)	121,153 (99.5)		4502 (99.0)	144,627 (99.4)		2277 (98.1)	146,852 (99.4)		26,207 (98.3)	122,922 (99.6)		9559 (97.6)	139,570 (99.5)	
70-79 yrs, ICU	409 (1.7)	316 (0.6)	<0.01	269 (1.7)	456 (0.8)	<0.01	42 (1.2)	683 (1.0)	0.10	31 (2.2)	694 (0.9)	<0.01	391 (2.4)	334 (0.6)	<0.01	140 (3.3)	585 (0.8)	<0.01
70-79 yrs, No ICU	24,069 (98.3)	50,195 (99.4)		15,989 (98.4)	58,275 (99.2)		3363 (98.8)	70,901 (99.1)		1402 (97.8)	72,862 (99.1)		15,983 (97.6)	58,281 (99.4)		4100 (96.7)	70,164 (99.2)	
80-89 yrs, ICU	189 (2.0)	116 (0.7)	<0.01	102 (1.9)	203 (1.0)	<0.01	30 (1.8)	275 (1.1)	0.01	15 (2.4)	290 (1.1)	<0.01	176 (2.6)	129 (0.7)	<0.01	52 (4.0)	253 (1.0)	<0.01
80-89 yrs, No ICU	9,424 (98.0)	17,033 (99.3)		5200 (98.1)	21,257 (99.1)		1677 (98.2)	24,780 (98.9)		610 (97.6)	25,847 (98.9)		6607 (97.4)	19,850 (99.4)		1247 (96.0)	25,210 (99.0)	
90-99 yrs, ICU	27 (2.3)	20 (0.7)	<0.01	8 (1.7)	39 (1.1)	0.29	4 (1.4)	43 (1.2)	0.57	2 (2.9)	45 (1.2)	0.20	25 (3.0)	22 (0.7)	<0.01	6 (5.4)	41 (1.1)	<0.01
90-99 yrs, No ICU	1158 (97.7)	2766 (99.3)		470 (98.3)	3454 (98.9)		273 (98.6)	3651 (98.8)		67 (97.1)	3857 (98.9)		811 (97.0)	3113 (99.3)		105 (94.6)	3819 (98.9)	
100-109 yrs, ICU	1 (1.7)	4 (1.4)	1.00	1 (4.4)	4 (1.2)	0.29	0	5 (1.5)	1.00	0	5 (1.5)	1.00	1 (3.3)	4 (1.3)	0.36	0	5 (1.5)	1.00
100-109 yrs, No ICU	58 (98.3)	287 (98.6)		22 (95.7)	323 (98.8)		19 (100.0)	326 (98.5)		4 (100.0)	341 (98.6)		29 (96.7)	316 (98.8)		6 (100.0)	339 (98.6)	
110-120 yrs, ICU	1 (3.7)	1(0.6)	0.26	0	2 (1.2)	1.00	0	2 (1.1)	1.00	NA	2 (1.0)	NA	1 (7.7)	1 (0.6)	0.13	0	2 (1.1)	1.00
110-120 yrs, No ICU	26 (96.3)	164 (99.4)		13 (100.0)	177 (98.9)		10 (100.0)	180 (98.9)		NA	190 (99.0)		12 (92.3)	178 (99.4)		5 (100.0)	185 (98.9)	

Abbreviations: CVD (cardiovascular disease including hypertension), HTN (hypertension), ICU (intensive care unit)

Supplementary Table 5 - Proportion of deaths with preexisting conditions by age group

Mortality by age groups	Cardiovascular Disease			Diabetes			Lung Disease			Kidney Disease			Hypertension			Obesity		
	Yes, n (%)	No, n (%)	*p* value	Yes, n (%)	No, n (%)	*p* value	Yes, n (%)	No, n (%)	*p* value	Yes, n (%)	No, n (%)	*p* value	Yes, n (%)	No, n (%)	*p* value	Yes, n (%)	No, n (%)	*p* value
Overall, deaths	12,074 (7.0)	15,539 (0.6)		8548 (7.6)	19,065 (0.7)		1888 (4.2)	25,725 (0.9)		1733 (15.8)	25,880 (0.9)		8238 (7.3)	19,375 (0.7)		4126 (4.3)	23,487 (0.9)	
Overall, alive	160,558 (93.0)	2608085 (99.4)	<0.01	103,437 (92.4)	2,665,206 (99.3)	<0.01	43,146 (95.8)	2725497 (99.1)	<0.01	9238 (84.2)	2,759,405 (99.1)	<0.01	104,144 (92.7)	2,664,499 (99.3)	<0.01	91,196 (95.7)	2677447 (99.1)	<0.01
0-9 yrs deaths	15 (2.9)	143 (0.2)	<0.01	2 (1.2)	156 (1.3)	0.07	6 (0.4)	152 (0.2)	0.18	0	158 (0.3)	1.00	4 (5.0)	154 (0.2)	<0.01	2 (0.7)	156 (0.3)	0.14
0-9 yrs alive	508 (97.1)	62,947 (99.8)		165 (98.8)	63,290 (99.8)		1440 (99.6)	62,015 (99.8)		79 (100.0)	63,376 (99.8)		76 (95.0)	63,379 (99.8)		267 (99.3)	63,188 (99.8)	
10-19 yrs, deaths	5 (0.8)	78 (0.1)	<0.01	4 (0.9)	79 (0.1)	<0.01	8 (0.3)	75 (0.1)	<0.01	2 (1.1)	81 (0.1)	0.01	1 (0.4)	82 (0.1)	0.17	7 (0.4)	76 (0.1)	<0.01
10-19 yrs, alive	610 (99.2)	107,949 (99.9)		442 (99.1)	108,117 (99.9)		2929 (99.7)	105,630 (99.9)		179 (98.9)	108,380 (99.9)		236 (99.6)	108,323 (99.9)		1950 (99.6)	106,609 (99.9)	
20-29 yrs, deaths	60 (1.3)	315 (0.1)	<0.01	43 (2.0)	332 (0.1)	<0.01	20 (0.3)	355 (0.1)	<0.01	50 (5.7)	325 (0.1)	<0.01	51 (1.7)	324 (0.1)	<0.01	59 (0.4)	316 (0.1)	<0.01
20-29 yrs, alive	4624 (98.7)	367,833 (99.9)		2136 (98.0)	370,321 (99.9)		7883 (99.8)	364,574 (99.9)		822 (94.3)	371,635 (99.9)		2884 (98.3)	369,573 (99.9)		13,971 (99.6)	358,486 (99.9)	
30-39 yrs, deaths	194 (1.5)	844 (0.2)	<0.01	127 (1.7)	911 (0.2)	<0.01	35 (0.4)	1003 (0.2)	<0.01	94 (7.6)	944 (0.2)	<0.01	140 (1.7)	898 (0.2)	<0.01	231 (1.1)	807 (0.2)	<0.01
30-39 yrs, alive	13,124 (98.5)	424,960 (99.8)		7203 (98.3)	430,881 (99.8)		8053 (99.6)	430,031 (99.8)		1146 (92.4)	436,938 (99.8)		8101 (98.3)	429,983 (99.8)		21,821 (99.0)	416,263 (99.8)	

40-49 yrs, deaths	736 (2.5)	11,890 (0.5)	<0.01	662 (3.4)	1964 (0.6)	<0.01	90 (1.2)	2536 (0.7)	<0.01	170 (11.0)	2456 (0.7)	<0.01	564 (2.9)	2062 (0.6)	<0.01	594 (2.6)	2032 (0.6)	<0.01
40-49 yrs, alive	29,099 (97.5)	347,455 (99.5)		18 701 (96.6)	357,853 (99.5)		7409 (98.8)	369,145 (99.3)		1370 (89.0)	375,184 (99.4)		19,205 (97.2)	357,349 (99.4)		22,357 (97.4)	354,197 (99.4)	
50-59 yrs, deaths	1838 (4.1)	2993 (1.3)	<0.01	1603 (5.2)	3228 (1.3)	<0.01	223 (3.7)	4608 (1.7)	<0.01	300 (13.3)	4531 (1.6)	<0.01	1413 (4.7)	3418 (1.4)	<0.01	981 (5.4)	3850 (1.5)	<0.01
50-59 yrs, alive	42,632 (95.9)	232,716 (98.7)		29,508 (94.9)	245,840 (98.7)		5866 (96.3)	269,482 (98.3)		1950 (86.7)	273,398 (98.4)		28,735 (95.3)	246,613 (98.6)		17,336 (94.6)	258,012 (98.5)	
60-69 yrs, deaths	3021 (7.8)	3471 (3.1)	<0.01	2593 (9.1)	3899 (3.2)		402 (8.8)	6090 (4.2)	<0.01	428 (18.4)	6064 (4.1)	<0.01	2420 (9.1)	4072 (3.3)	<0.01	1070 (10.9)	5422 (3.9)	<0.01
60-69 yrs, alive	35,810 (92.2)	107,768 (96.9)		25,762 (90.9)	117 816 (96.8)		4145 (91.2)	139,433 (95.8)		1893 (81.6)	141,685 (95.9)		24,230 (90.9)	119,348 (96.7)		8721 (89.1)	134,857 (96.1)	
70-79 yrs, deaths	3090 (12.6)	3045 (6.0)	<0.01	2290 (14.1)	3845 (6.6)	<0.01	461 (13.5)	5674 (7.9)	<0.01	335 (23.4)	5800 (7.9)	<0.01	2324 (14.2)	3 811 (6.5)	<0.01	750 (17.7)	5385 (7.6)	<0.01
70-79 years, alive	21,388 (87.4)	47,466 (94.0)		13,968 (85.9)	54,886 (93.5)		2944 (86.5)	65,910 (92.1)		1098 (76.6)	67,756 (92.1)		14,050 (85.8)	54,804 (93.5)		3490 (82.3)	65,364 (92.4)	
80-89 yrs, deaths	1548 (16.1)	1688 (9.8)	<0.01	968 (18.3)	2268 (10.6)	<0.01	303 (17.8)	2933 (11.7)	<0.01	148 (23.7)	3088 (11.8)	<0.01	1094 (16.1)	2142 (10.7)	<0.01	265 (20.4)	2971 (11.7)	<0.01
80-89 yrs, alive	8065 (83.9)	15,461 (90.2)		4334 (81.7)	19,192 (89.4)		1404 (82.3)	22,122 (88.3)		477 (76.3)	23,049 (88.2)		5689 (83.9)	17,837 (89.3)		1034 (79.6)	22,492 (88.3)	
90-99 yrs, deaths	222 (18.7)	269 (9.7)	<0.01	110 (23.0)	381 (10.9)	<0.01	49 (17.7)	442 (12.0)	0.01	16 (23.2)	475 (12.2)	0.01	170 (20.3)	321 (10.2)	<0.01	24 (21.6)	467 (12.1)	<0.01
90-99 yrs, alive	963 (81.3)	2517 (90.3)		368 (77.0)	3112 (89.1)		228 (82.3)	3252 (88.0)		53 (76.8)	3427 (87.8)		666 (79.7)	2814 (89.8)		87 (78.4)	3393 (87.9)	
100-109 yrs, deaths	8 (13.6)	17 (5.8)	0.05	1 (4.4)	24 (7.3)	1.00	2 (10.5)	23 (7.0)	0.64	0	25 (7.2)	1.00	1 (3.3)	24 (7.5)	0.71	0	25 (7.3)	1.00

100-109 yrs, alive	51 (86.4)	274 (94.2)		22 (95.7)	303 (92.7)		17 (89.5)	308 (93.1)		4 (100.0)	321 (92.8)		29 (96.7)	296 (92.5)		6 (100.0)	319 (92.7)	
110-120 yrs, deaths	4 (14.8)	10 (6.1)	0.12	1 (7.7)	13 (7.3)	1.00	0	14 (7.7)	1.00	NA	14 (7.3)	NA	3 (23.1)	11 (6.2)	0.06	1 (20.0)	13 (7.0)	0.32
110-120 yrs, alive	23 (85.2)	155 (93.9)		12 (92.3)	166 (92.7)		10 (100.0)	168 (92.3)		NA	178 (92.7)		10 (76.9)	168 (93.9)		4 (80.0)	174 (93.1)	

Abbreviations: CVD (cardiovascular disease including hypertension), HTN (hypertension), ICU (intensive care unit)

Supplementary Table 6- Unadjusted and adjusted odds ratios for hospitalization for each preexisting condition by age group–multivariable logistic regression

Age groups	Cardiovascular diseases		Lung Diseases		Diabetes		Kidney diseases		Obesity		Hypertension	
	OR (95% CI)	aOR (95% CI)	OR (95% CI)	aOR (95% CI)	OR (95% CI)	aOR (95% CI)	OR (95% CI)	aOR (95% CI)	OR (95% CI)	aOR (95% CI)	OR (95% CI)	aOR (95% CI)
Overall	6.8 (6.7-6.9)	1.7 (1.7-1.7)	2.9 (2.8-3.0)	1.9 (1.8-1.9)	8.0 (7.9-8.2)	2.2 (2.1-2.2)	18.9 (18.2-19.6)	5.5 (5.2-5.7)	4.1 (4.0-4.1)	1.7 (1.6-1.7)	9.7 (9.5-9.8)	1.5 (1.4-1.5)
0-9 yrs	7.0 (5.8-8.4)	4.4 (3.5-5.5)	2.1 (1.8-2.5)	1.5 (1.3-1.8)	2.5 (1.7-3.8)	1.3 (0.8-2.2)	4.3 (2.6-7.3)	1.3 (0.7-2.3)	2.3 (1.6-3.2)	0.6 (0.4-0.8)	5.5 (3.4-9.0)	1.3 (0.8-2.2)
10-19 yrs	6.7 (5.2-8.7)	3.6 (2.7-4.8)	2.3 (1.9-2.8)	1.9 (1.5-2.3)	9.9 (7.6-12.9)	5.7 (4.3-7.7)	36.5 (27.0-49.3)	15.8 (11.4-22.1)	3.2 (2.6-3.9)	1.4 (1.1-1.7)	10.6 (7.5-15.0)	3.0 (2.0-4.4)
20-29 yrs	8.4 (7.5-9.3)	4.4 (3.9-5.0)	1.8 (1.5-2.1)	1.5 (1.2-1.7)	11.2 (9.8-12.9)	4.9 (4.2-5.7)	40.9 (35.3-47.4)	14.9 (12.6-17.6)	3.3 (3.0-3.7)	1.4 (1.3-1.6)	12.3 (11.0-13.9)	4.5 (4.0-5.1)

30-39 yrs	4.9 (4.6-5.3)	2.4 (2.2-2.6)	1.9 (1.7-2.2)	1.6 (1.4-1.8)	7.1 (6.6-7.8)	3.3 (3.0-3.6)	31.7 (28.1-35.8)	11.9 (10.4-13.7)	4.4 (4.1-4.6)	1.7 (1.6-1.8)	7.1 (6.6-7.7)	2.2 (2.1-2.4)
40-49 yrs	3.8 (3.6-4.0)	1.8 (1.7-1.9)	2.0 (1.8-2.2)	1.7 (1.5-1.9)	6.2 (5.9-6.5)	3.1 (3.0-3.3)	19.4 (17.4-21.6)	7.1 (6.3-8.0)	4.5 (4.3-4.7)	1.7 (1.7-1.8)	5.3 (5.1-5.6)	1.6 (1.5-1.7)
50-59 yrs	3.4 (3.3-3.5)	1.7 (1.6-1.8)	2.2 (2.0-2.3)	1.9 (1.7-2.0)	4.7 (4.5-4.9)	2.5 (2.4-2.5)	14.3 (13.2-15.6)	5.3 (4.8-5.8)	4.1 (3.9-4.3)	1.6 (1.6-1.7)	4.7 (4.5-4.9)	1.5 (1.5-1.6)
60-69 yrs	3.1 (3.0-3.2)	1.6 (1.5-1.6)	2.4 (2.2-2.6)	2.1 (1.9-2.3)	3.6 (3.4-3.7)	2.0 (1.9-2.1)	10.2 (9.4-11.1)	4.1 (3.7-4.5)	4.1 (3.9-4.3)	1.7 (1.6-1.8)	4.4 (4.3-4.6)	1.4 (1.3-1.4)
70-79 yrs	3.1 (3.0-3.2)	1.6 (1.5-1.6)	2.5 (2.3-2.7)	2.1 (2.0-2.3)	3.0 (2.9-3.2)	1.6 (1.6-1.7)	6.8 (6.1-7.6)	2.9 (2.5-3.2)	4.5 (4.2-4.8)	1.7 (1.6-1.8)	4.9 (4.7-5.1)	1.3 (1.2-1.3)
80-89 yrs	3.2 (3 .0-3.4)	1.5 (1.4-1.6)	2.9 (2.6-3.2)	1.8 (1.6-2.1)	2.7 (2.6-2.9)	1.5 (1.4-1.6)	4.8 (4.0-5.6)	2.0 (1.7-2.4)	3.9 (3.5-4.3)	1.4 (1.2-1.6)	5.4 (5.1-5.7)	1.3 (1.2-1.4)
90-99 yrs	3.7 (3.1-4.3)	1.4 (1.2-1.7)	2.8 (2.2-3.6)	1.7 (1.2-2.3)	2.8 (2.3-3.4)	1.4 (1.1-1.8)	3.9 (2.4-6.4)	1.1 (0.7-1.9)	4.2 (2.9-6.2)	1.3 (0.9-2.0)	6.4 (5.4-7.5)	1.4 (1.1-1.7)
100-109 yrs	4.4 (2.1-9.1)	3.9 (1.5-9.7)	4.6 (1.6-13.1)	3.7 (1.1-12.5)	3.5 (1.3-9.5)	0.8 (0.2-3.1)	1 (NA)	1 (NA)	4.6 (0.8-25.8)	1.5 (0.2-10.5)	9.9 (4.3-22.8)	3.3 (1.2-8.9)
110-120 yrs	3.6 (1.2-10.7)	3.7 (1.0-12.8)	4.8 (1.1-20.4)	3.0 (0.7-14.1)	8.0 (2.3-27.9)	8.0 (1.8-35.4)	1 (NA)	1 (NA)	2.5 (0.3-23.7)	0.7 (0.1-8.9)	11.9 (3.5-41.1)	5.5 (1.4-21.1)

CVD adjusted for gender, country, diabetes and obesity. Lung diseases adjusted for gender, country and obesity. Diabetes adjusted for gender, country, CVD and obesity. Kidney diseases adjusted for gender, country, CVD, diabetes and obesity. Obesity adjusted forgender, country, CVD and diabetes. HTN adjusted for gender, country, diabetes and obesity.

Supplementary Table 7- Unadjusted and adjusted odds ratios for ICU for each preexisting condition by age group–multivariable logistic regression

Age groups	Cardiovascular diseases		Lung Diseases		Diabetes		Kidney Diseases		Obesity		Hypertension	
	OR (95% CI)	aOR (95% CI)	OR (95% CI)	aOR (95% CI)	OR (95% CI)	aOR (95% CI)	OR (95% CI)	aOR (95% CI)	OR (95% CI)	aOR (95% CI)	OR (95% CI)	aOR (95% CI)
Overall	6.3 (5.9-6.7)	1.4 (1.3-1.5)	2.1 (1.8-2.4)	1.1 (0.9-1.3)	7.0 (6.5-7.5)	1.7 (1.5-1.8)	7.5 (6.4-8.7)	1.4 (1.2-1.7)	5.6 (5.2-6.0)	2.2 (2.1-2.4)	8.9 (8.4-9.5)	1.3 (1.2-1.4)
0-9 yrs	5.1 (2.7-9.6)	2.7 (1.4-5.1)	0.3 (0.1-1.4)	0.2 (0.1-1.0)	1.5 (0.2-10.9)	0.9 (0.1-6.7)	3.2 (0.4-23.3)	1.1 (0.1-8.1)	NA	NA	3.2 (0.4-23.0)	1.1 (0.1-7.9)
10-19 yrs	7.2 (2.3-23.1)	3.1 (0.9-11.0)	1.0 (0.2-4.0)	0.7 (0.2-3.0)	10.0 (3.1-31.9)	4.1 (1.1-14.8)	8.0 (1.1-58.1)	1.8 (0.2-15.3)	8.3 (4.3-16.2)	3.7 (1.9-7.4)	6.1 (0.8-44.2)	1.2 (0.1-10.1)
20-29 yrs	6.2 (3.4-11.1)	1.8 (1.0-3.5)	1.7 (0.8-3.9)	1.3 (0.6-3.0)	21.0 (12.8-34.3)	10.0 (5.8-17.3)	19.0 (8.9-40.7)	5.3 (2.2-12.7)	6.2 (4.2-9.1)	2.6 (1.7-3.8)	8.2 (4.3-15.5)	1.6 (0.8-3.2)
30-39 yrs	5.4 (3.9-7.4)	2.0 (1.4-2.8)	1.6 (0.8-3.2)	1.3 (0.7-2.5)	9.4 (6.7-13.0)	3.8 (2.7-5.4)	20.1 (12.1-33.4)	5.5 (3.1-9.6)	7.6 (5.9-9.8)	2.8 (2.2-3.6)	8.1 (5.8-11.3)	1.9 (1.3-2.7)
40-49 yrs	3.3 (2.6-4.0)	1.2 (1.0-1.6)	2.3 (1.5-3.5)	1.8 (1.2-2.8)	6.0 (4.9-7.3)	2.7 (2.2-3.4)	9.7 (6.1-15.4)	3.0 (1.8-4.8)	7.4 (6.1-8.9)	2.9 (2.4-3.5)	4.6 (3.7-5.7)	1.1 (0.9-1.4)
50-59 yrs	2.9 (2.5-3.3)	1.3 (1.1-1.5)	1.7 (1.2-2.5)	1.4 (1.0-2.0)	4.5 (3.9-5.2)	2.3 (2.0-2.7)	5.0 (3.5-7.2)	1.6 (1.1-2.3)	5.0 (4.2-5.8)	2.1 (1.7-2.4)	4.7 (4.5-4.9)	1.5 (1.5-1.6)
60-69 yrs	2.9 (2.5-3.3)	1.4 (1.2-1.7)	1.6 (1.2-2.2)	1.2 (0.9-1.6)	2.9 (2.6-3.3)	1.5 (1.3-1.7)	3.2 (2.3-4.3)	1.2 (0.9-1.6)	4.1 (3.9-4.3)	1.7 (1.6-1.8)	4.2 (3.7-4.7)	1.3 (1.2-1.5)
70-79 yrs	2.7 (2.3-3.1)	1.4 (1.2-1.6)	1.3 (0.9-1.8)	0.9 (0.7-1.3)	2.2 (1.8-2.5)	1.1 (0.9-1.3)	2.3 (1.6-3.3)	1.0 (0.7-1.5)	4.5 (4.2-4.8)	1.7 (1.6-1.8)	4.3 (3.7-4.9)	1.4 (1.2-1.6)

80-89 yrs	2.9 (2.3-3.7)	1.5 (1.2-1.9)	1.6 (1.1-2.4)	1.0 (0.7-1.4)	2.1 (1.6-2.6)	1.1 (0.9-1.4)	2.2 (1.3-3.7)	1.1 (0.6-1.8)	4.2 (3.1-5.6)	2.0 (1.5-2.7)	4.1 (3.3-5.2)	1.4 (1.1-1.7)
90-99 yrs	3.2 (1.8-5.8)	1.6 (0.9-3.0)	1.2 (0.4-3.5)	0.8 (0.3-2.1)	1.5 (0.7-3.2)	0.7 (0.3-1.6)	2.6 (0.6-10.8)	1.1 (0.2-4.6)	5.3 (2.2-12.8)	2.6 (1.1-6.5)	4.4 (2.4-7.8)	1.6 (0.9-2.9)
100-109 yrs	1.2 (0.1-11.3)	0.5 (0.0-8.1)	1 (NA)	1 (NA)	3.7 (0.4-34.3)	4.2 (0.3-70.2)	1 (NA)	1 (NA)	1 (NA)	1 (NA)	2.7 (0.3-25.2)	0.6 (0.0-11.1)
110-120 yrs	6.3 (0.4-104.0)	7.5 (0.4-132.7)	1 (NA)	1 (NA)	1 (NA)	1 (NA)	1 (NA)	1 (NA)	1 (NA)	1 (NA)	14.8 (0.9-252.1)	7.5 (0.4-132.7)

CVD adjusted for gender, country, diabetes and obesity. Lung diseases adjusted for gender, country and obesity. Diabetes adjusted for gender, country, CVD and obesity. Kidney diseases adjusted for gender, country, CVD, diabetes and obesity. Obesity adjusted for gender, country, CVD and diabetes. HTN adjusted for gender, country, diabetes and obesity.

Table 8 - Unadjusted and adjusted odds ratios for mortality for each preexisting condition by age group–multivariable logistic regression

Age groups	Cardiovascular diseases		Lung Diseases		Diabetes		Kidney Diseases		Obesity		Hypertension	
	OR (95% CI)	aOR (95% CI)	OR (95% CI)	aOR (95% CI)	OR (95% CI)	aOR (95% CI)	OR (95% CI)	aOR (95% CI)	OR (95% CI)	aOR (95% CI)	OR (95% CI)	aOR (95% CI)
Overall	8.4 (8.2-8.7)	1.7 (1.6-1.7)	3.1 (3.0-3.3)	1.6 (1.5-1.7)	7.9 (7.6-8.1)	2.0 (1.9-2.0)	13.7 (13.0-14.5)	2.7 (2.6-2.9)	3.5 (3.4-3.7)	1.9 (1.8-2.0)	7.5 (7.3-7.7)	1.3 (1.3-1.4)
0-9 yrs, n (%)	13.0 (7.6-22.3)	7.9 (4.5-13.8)	1.7 (0.8-3.9)	1.3 (0.6-3.0)	4.9 (1.2-20.0)	1.5 (0.3-6.6)	1 (NA)	1 (NA)	3.0 (0.7-12.3)	1.0 (0.2-4.2)	21.7 (7.8-59.9)	7.7 (2.5-24.0)

10-19 yrs, n (%)	11.3 (4.6-28.1)	6.2 (2.2-17.6)	3.8 (1.9-8.0)	3.5 (1.7-7.4)	12.4 (4.5-34.0)	5.5 (1.7-17.6)	15.0 (2.6-61.2)	3.9 (0.7-20.6)	5.0 (2.3-10.9)	3.7 (1.6-8.7)	5.6 (0.8-40.4)	1.4 (0.2-12.3)
20-29 yrs, n (%)	15.2 (11.5-20.0)	6.9 (4.9-9.6)	2.6 (1.7-4.1)	2.3 (1.5-3.6)	22.5 (16.3-30.9)	8.3 (5.7-12.1)	69.6 (51.2-94.4)	22.6 (15.1-33.9)	4.8 (3.6-6.3)	2.6 (1.9-3.6)	20.2 (15.0-27.2)	7.4 (5.1-10.7)
30-39 yrs, n (%)	7.4 (6.4-8.7)	3.7 (3.1-4.4)	1.9 (1.3-2.6)	1.7 (1.2-2.3)	8.3 (6.9-10.1)	3.6 (2.9-4.4)	38.0 (30.5-47.3)	12.6 (9.7-16.3)	5.5 (4.7-6.3)	2.8 (2.4-3.3)	8.3 (6.9-9.9)	2.9 (2.3-3.5)
40-49 yrs, n (%)	4.6 (4.3-5.1)	2.3 (2.1-2.6)	1.8 (1.4-2.2)	1.6 (1.3-2.0)	6.4 (5.9-7.1)	3.3 (3.0-3.6)	19.0 (16.1-22.3)	5.9 (4.9-7.1)	4.6 (4.2-5.1)	2.2 (2.0-2.4)	5.1 (4.6-5.6)	1.8 (1.6-2.0)
50-59 yrs, n (%)	3.4 (3.2-3.6)	1.9 (1.8-2.1)	2.2 (1.9-2.5)	2.0 (1.7-2.3)	4.1 (3.9-4.4)	2.4 (2.2-2.5)	9.3 (8.2-10.5)	3.3 (2.9-3.8)	3.8 (3.5-4.1)	1.9 (1.8-2.1)	3.5 (3.3-3.8)	1.5 (1.4-1.6)
60-69 yrs, n (%)	2.6 (2.5-2.8)	1.6 (1.5-1.7)	2.2 (2.0-2.5)	1.9 (1.7-2.1)	3.0 (2.9-3.2)	2.0 (1.8-2.1)	5.3 (4.7-5.9)	2.3 (2.0-2.6)	3.1 (2.8-3.3)	1.7 (1.5-1.8)	2.9 (2.8-3.1)	1.3 (1.2-1.4)
70-79 yrs, n (%)	2.3 (2.1-2.4)	1.5 (1.4-1.6)	1.8 (1.6-2.0)	1.6 (1.4-1.8)	2.3 (2.2-2.5)	1.6 (1.5-1.7)	3.6 (3.1-4.0)	1.9 (1.6-2.1)	2.6 (2.4-2.8)	1.5 (1.4-1.7)	2.4 (2.3-2.5)	1.2 (1.1-1.3)
80-89 yrs, n (%)	1.8 (1.6-1.9)	1.4 (1.2-1.5)	1.6 (1.4-1.9)	1.4 (1.2-1.6)	1.9 (1.7-2.1)	1.5 (1.4-1.7)	2.3 (1.9-2.8)	1.5 (1.3-1.9)	1.9 (1.7-2.2)	1.4 (1.2-1.6)	1.6 (1.5-1.7)	1.0 (0.9-1.1)
90-99 yrs , n (%)	2.2 (1.8-2.6)	1.5 (1.2-1.9)	1.6 (1.1-2.2)	1.3 (0.9-1.8)	2.4 (1.9-3.1)	1.8 (1.4-2.3)	2.2 (1.2-3.8)	1.1 (0.6-2.0)	2.0 (1.3-3.2)	1.2 (0.8-2.0)	2.2 (1.8-2.7)	1.4 (1.1-1.8)

100-109 yrs, n (%)	2.5 (1.0-6.2)	3.5 (1.3-9.1)	1.6 (0.3-7.2)	2.1 (0.4-10.0)	0.6 (0.1-4.4)	0.4 (0.1-3.9)	1 (NA)	1 (NA)	1 (NA)	1 (NA)	0.4 (0.1-3.3)	1.5 (0.1-16.6)
110-119 yrs, n (%)	2.7 (0.8-9.3)	2.8 (0.8-9.9)	1 (NA)	1 (NA)	1.1 (0.1-8.8)	0.8 (0.1-7.1)	1 (NA)	1 (NA)	3.3 (0.3-32.2)	3.6 (0.3-39.2)	4.6 (1.1-19.1)	8.1 (1.4-46.6)

CVD adjusted for gender, country, diabetes and obesity. Lung diseases adjusted for gender, country and obesity. Diabetes adjusted for gender, country, CVD and obesity. Kidney diseases adjusted for gender, country, CVD, diabetes and obesity. Obesity adjusted for gender, country, CVD and diabetes. HTN adjusted for gender, country, diabetes and obesity.
