# Supplementary material for: Association Between Pre-Existing Conditions and COVID-19 Hospitalization, Intensive Care Services, and Mortality: A Cross-Sectional Analysis of an International Global Health Data Repository
Source: Pathogens. 2025 Sep 11;14(9):917. doi: 10.3390/pathogens14090917 (PMC12472582; doi:10.3390/pathogens14090917)
Supplement: Supplementary file 1 [file pathogens-14-00917-s001.zip › pathogens-3816492-supplementary/pathogens-3816492-supplementary-2.pdf]

```
***tab preexistingconditionsvalues

***organizing preexistingconditionsvalues

***CVD from pre-existing conditions
gen long cvd=0
gen long cvd_d = 0
foreach disease in HYPERTENSION "CARDIOVASCULAR SYSTEM DISEASE" "CARDIOVASCULAR DISEASE" "HYPERTENSION" "HEART DISEASE" "HIPERTENSIÓN" "HIPERTENSÃO" "HYPERTENSION ARTÉRIELLE" "CARDIOVASCULAIRE" "CARDIOPATÍA" "DOENÇA CARDÍACA" "CARDIOPATHIE" "PRESSÃO ALTA" "ALTA PRESIÓN SANGUÍNEA" "la hipertensión" {
replace cvd_d= cvd_d + 1 if strpos(upper(preexistingconditionsvalues), "`disease'") > 0
}
replace cvd=1 if cvd_d > 0
drop cvd_d
tab cvd

***CVD from notes
gen long cvd_d_notes= 0
foreach disease in HYPERTENSION "CARDIOVASCULAR SYSTEM DISEASE" "CARDIOVASCULAR DISEASE" "HYPERTENSION" "HEART DISEASE" "HIPERTENSIÓN" "HIPERTENSÃO" "HYPERTENSION ARTÉRIELLE" "CARDIOVASCULAIRE" "CARDIOPATÍA" "DOENÇA CARDÍACA" "CARDIOPATHIE" "PRESSÃO ALTA" "ALTA PRESIÓN SANGUÍNEA" "la hipertensión" {
replace cvd_d_notes= cvd_d_notes + 1 if strpos(upper(notes), "`disease'") > 0
}
replace cvd=1 if cvd_d_notes > 0
drop cvd_d_notes
tab cvd
```

***HTN from pre-existing conditions

gen long htn=0

gen long htn_d = 0

foreach disease in HYPERTENSION "HYPERTENSION" "HIGH BLOOD PRESSURE" "HIGH BP" "HIPERTENSIÓN" "HIPERTENSÃO" "HYPERTENSION ARTÉRIELLE" "PRESSÃO ALTA" "ALTA PRESIÓN SANGUÍNEA" "la hipertensión" {

replace htn_d= htn_d + 1 if strpos(upper(preexistingconditionsvalues), "`disease'") > 0

}

replace htn=1 if htn_d > 0

drop htn_d

tab htn

***HTN from notes

gen long htn_d_notes= 0

foreach disease in HYPERTENSION "HYPERTENSION" "HIGH BLOOD PRESSURE" "HIGH BP" "HIPERTENSIÓN" "HIPERTENSÃO" "HYPERTENSION ARTÉRIELLE" "PRESSÃO ALTA" "ALTA PRESIÓN SANGUÍNEA" "la hipertensión" {

replace htn_d_notes= htn_d_notes + 1 if strpos(upper(notes), "`disease'") > 0

}

replace htn=1 if htn_d_notes > 0

drop htn_d_notes

tab htn

***pregnancy from pre-existing conditions

gen long pregnancy=0

```
gen long pregnancy_d = 0

foreach disease in  "pregnancy" "high risk pregnancy" "recently gave birth" "gestating"
"expecting" "gravid" "grossesse" "gravidez" "el embarazo"{

replace pregnancy_d= pregnancy_d + 1 if strpos(lower(preexistingconditionsvalues), "`disease'")
> 0

}

replace pregnancy=1 if pregnancy_d > 0

drop pregnancy_d

tab pregnancy




***pregnancy from notes

gen long pregnancy_d_notes = 0

foreach disease in "pregnancy" "high risk pregnancy" "recently gave birth" "gestating"
"expecting" "gravid" "grossesse" "gravidez" "el embarazo" {

replace pregnancy_d_notes= pregnancy_d_notes + 1 if strpos(lower(notes), "`disease'") > 0

}

replace pregnancy_notes=1 if pregnancy_d_notes > 0

drop pregnancy_d_notes

tab pregnancy_notes


***add all the pregancies together

replace pregnancy =pregnancy_notes if pregnancy==.



***Lung diseases from pre-existing conditions

gen long lung=0
```

```
gen long lung_d = 0

foreach disease in asthma "lung disease" "chronic obstructive pulmonary disease" "respiratory system disease" "asma" "Doença pulmonar" "enfermedad pulmonar" "les maladies pulmonaires" "bronchopneumopathie chronique obstructive" "doença de obstrução pulmonar crônica" "enfermedad pulmonar obstructiva crónica" "respiratoria" "respiratorio" "respiratória" "respiratório" "respiratoire" "COPD" {

replace lung_d= lung_d + 1 if strpos(lower(preexistingconditionsvalues), "`disease'") > 0

}

replace lung=1 if lung_d > 0

drop lung_d

tab lung


***Lung diseases from notes

gen long lung_d_notes = 0

foreach disease in asthma "lung disease" "chronic obstructive pulmonary disease" "respiratory system disease" "asma" "Doença pulmonar" "enfermedad pulmonar" "les maladies pulmonaires" "bronchopneumopathie chronique obstructive" "doença de obstrução pulmonar crônica" "enfermedad pulmonar obstructiva crónica" "respiratoria" "respiratorio" "respiratória" "respiratório" "respiratoire" "COPD" {

replace lung_d_notes= lung_d_notes + 1 if strpos(lower(notes), "`disease'") > 0

}

replace lung=1 if lung_d_notes > 0

drop lung_d_notes

tab lung



***diabetes from pre-existing conditions

gen long diabetes=0
```

```
gen long diabetes_d = 0

foreach disease in diabetes "diabetes" "diabetes mellitus" "type 1 diabetes" "type 2 diabetes" "t1dm" "t2dm" "gestational diabetes" "diabète" "la diabetes" "diabetes tipo 2" "diabetes tipo 1" "diabetes gestacional" "diabète de type 2" "diabète de type 1" "diabète gestationnel" {

replace diabetes_d= diabetes_d + 1 if strpos(lower(preexistingconditionsvalues), "`disease'") > 0

}

replace diabetes=1 if diabetes_d > 0

drop diabetes_d

tab diabetes


***diabetes from notes

gen long diabetes_d_notes = 0

foreach disease in diabetes "diabetes" "diabetes mellitus" "type 1 diabetes" "type 2 diabetes" "t1dm" "t2dm" "gestational diabetes" "diabète" "la diabetes" "diabetes tipo 2" "diabetes tipo 1" "diabetes gestacional" "diabète de type 2" "diabète de type 1" "diabète gestationnel" {

replace diabetes_d_notes= diabetes_d_notes + 1 if strpos(lower(notes), "`disease'") > 0

}

replace diabetes=1 if diabetes_d_notes > 0

drop diabetes_d_notes

tab diabetes


***disease of metabolism from pre-existing conditions

gen long metabolism=0

gen long metabolism_d = 0
```

```
foreach disease in "disease of metabolism" "doença do metabolismo" "enfermedad del metabolismo" "maladie du métabolisme" {

replace metabolism_d= metabolism_d + 1 if strpos(lower(preexistingconditionsvalues), "`disease'") > 0

}

replace metabolism=1 if metabolism_d > 0

drop metabolism_d

tab metabolism


***disease of metabolism from notes

gen long metabolism_d_notes = 0

foreach disease in "disease of metabolism" "doença do metabolismo" "enfermedad del metabolismo" "maladie du métabolisme" {

replace metabolism_d_notes= metabolism_d_notes + 1 if strpos(lower(notes), "`disease'") > 0

}

replace metabolism=1 if metabolism_d_notes > 0

drop metabolism_d_notes

tab metabolism


***kidney diseases from pre-existing conditions

gen long kidney=0

gen long kidney_d = 0

foreach disease in "kidney disease" "chronic kidney disease" "renal disease" "un rein" "le rein" "maladie du rein" "maladie rénale" "doença renal" "enfermedad renal" "nefropatía" {

replace kidney_d= kidney_d + 1 if strpos(lower(preexistingconditionsvalues), "`disease'") > 0

}

replace kidney=1 if kidney_d > 0
```

```
drop kidney_d

tab kidney


***kidney diseases from notes

gen long kidney_d_notes = 0

foreach disease in "kidney disease" "chronic kidney disease" "renal disease" "un rein" "le rein"
"maladie du rein" "maladie rénale" "doença renal" "enfermedad renal" "nefropatía" {

replace kidney_d_notes= kidney_d_notes + 1 if strpos(lower(notes), "`disease'") > 0

}

replace kidney=1 if kidney_d_notes > 0

drop kidney_d_notes

tab kidney


***obesity from preexisting conditions

gen long obesity=0

gen long obesity_d = 0

foreach disease in "obesity" "obesity" "obésité" "la obesidad" "obesidade" "overweight" {

replace obesity_d= obesity_d + 1 if strpos(lower(preexistingconditionsvalues), "`disease'") > 0

}

replace obesity=1 if obesity_d > 0

drop obesity_d

tab obesity


***obesity from notes

gen long obesity_d_notes = 0

foreach disease in "obesity" "obésité" "la obesidad" "obesidade" "overweight" {
```

```
replace obesity_d_notes= obesity_d_notes + 1 if strpos(lower(notes), "`disease'") > 0

}

replace obesity=1 if obesity_d_notes > 0

drop obesity_d_notes

tab obesity


***other conditions in the "pre-existing"

**2 liver diseases, 1 down syndrom


***extract the outcomes from the notes variable (death, icu, discharged/recovered), japan

*death

gen long death_d = 0

foreach disease in "died" died "death" "死" "死亡しました" "fallecida" "fallecido" "décédés" {

replace death_d = death_d + 1 if strpos(lower(notes), "`disease'") > 0

}

replace death=1 if death_d  > 0

drop death_d


*icu

gen long icu_d = 0

foreach disease in "critical" {

replace icu_d = icu_d + 1 if strpos(lower(notes), "`disease'") > 0

}

replace icu=1 if icu_d  > 0

drop icu_d
```

```
*hosp

gen long hosp_d = 0

foreach disease in "died at a hospital" "hospitalized" "Patient at hospital of" "patients at
hospital 仁惠病院" {

replace hosp_d = hosp_d + 1 if strpos(lower(notes), "`disease'") > 0

}

replace hosp=1 if hosp_d  > 0

drop hosp_d


*recovered

gen long recover_d = 0

foreach disease in "recovered" "discharged"  {

replace recover_d = recover_d + 1 if strpos(lower(notes), "`disease'") > 0

}

replace death=0 if recover_d  > 0

drop recover_d


***Quick analysis ((((NEED TO BE RUN FIRST))))

encode eventsoutcomevalue, gen(outcome)

replace outcome=0 if outcome==.

encode eventsicuadmissionvalue, gen(icu)

replace icu=0 if icu==.

drop symp

encode symptomsstatus, gen(symp)


encode eventshospitaladmissionvalue, gen(hosp)
```

```
replace hosp=0 if hosp==.

***the following variables and changes are not part of the data, it should be added
rename demographicsagerangeend age
rename demographicsethnicity ethnicity
rename demographicsgender gender
rename demographicsnationalities nationality
rename demographicsoccupation occupation
rename eventsconfirmeddate confirmdate
rename eventsonsetsymptomsvalue symptomstype
rename locationcountry country

***reorgaize the "outcome" variable
gen death=0
replace death=1 if outcome==1
replace icu=1 if outcome==7
replace hosp=1 if outcome==6
replace death=0 if outcome==2|outcome==4|outcome==5|outcome==6
replace death=. if outcome==3


***month of diagnosis (confirmdate), THE FINAL VARIABLE IS "diagnosis"
gen date=date( eventsconfirmeddate ,"YMD")
format date %tdnn/dd/CCYY
gen month=month( date )
gen year=year( date )
```

gen diagnosis=1 if month==1 & year==2020

replace diagnosis=2 if month==2 & year==2020

replace diagnosis=3 if month==3 & year==2020

replace diagnosis=4 if month==4 & year==2020

replace diagnosis=5 if month==5 & year==2020

replace diagnosis=6 if month==6 & year==2020

replace diagnosis=7 if month==7 & year==2020

replace diagnosis=8 if month==8 & year==2020

replace diagnosis=9 if month==9 & year==2020

replace diagnosis=10 if month==10 & year==2020

replace diagnosis=11 if month==11 & year==2020

replace diagnosis=12 if month==12 & year==2020

replace diagnosis=13 if month==1 & year==2021

replace diagnosis=14 if month==2 & year==2021

replace diagnosis=15 if month==3 & year==2021

replace diagnosis=16 if month==4 & year==2021

replace diagnosis=17 if month==5 & year==2021

replace diagnosis=18 if month==6 & year==2021

***age groups:

***create age groups for adults (0-17, 18-29, 30-39, 40-49, 50-59,...)

recode age (0/17=0)(18/29=1) (30/39=2) (40/49=3) (50/59=4) (60/69=5) (70/79=6) (80/89=7) (90/99=8) (100/109=9) (110/119=10) (120/129=11) (130/139=12)..., gen (adultsagegroups)

***table 1 descriptive:::

```
***tab dempgraphics

***tab preexistingconditions

***tab outcomes

***tab month of diagnosis

tab country

sum age, detail

tab hosp

tab icu

tab death

tab symp */




/***table1--characteristics of cases in the global health data set as of “Date”:

log using "D:\Preg COVID\Log files\Table 1 analysis 25may2021.log", replace

tab gender hosp, row mi

tab gender icu, row mi

tab gender death, row mi


tab age10 hosp, row mi

tab age10 icu, row mi

tab age10 death, row mi


tab year hosp, row mi

tab year icu, row mi

tab year death, row mi
```

```
tab diagnosis hosp, row mi

tab diagnosis icu, row mi

tab diagnosis death, row mi


/*excel

tab countrymatch hosp, row mi

tab countrymatch icu, row mi

tab countrymatch death, row mi*/



log close






keep if countrymatch==19|countrymatch==33 |countrymatch==25|
countrymatch==58|countrymatch== 92|countrymatch== 46|countrymatch==
82|countrymatch== 83|countrymatch== 119|countrymatch== 136

save "countries reporting comorbidities"


***table2--countries reporting pre-existing conditions:

use "D:\Preg COVID\countries reporting comorbidities.dta"

log using "D:\Preg COVID\Log files\Table 2 analysis 25may2021.log", replace


tab countrymatch cvd, row mi

tab countrymatch htn, row mi
```

```
tab countrymatch diabetes, row mi

tab countrymatch lung, row mi

tab countrymatch kidney, row mi

tab countrymatch obesity , row mi

tab countrymatch metabolism, row mi

tab countrymatch pregnancy, row mi

tab countrymatch hosp, row mi

tab countrymatch icu, row mi

tab countrymatch death, row mi


log close */


*******TABLE 3 characteristics and outcomes in people with pre-existing conditions

keep if countrymatch==19|countrymatch==33|countrymatch==82

save "D:\Preg COVID\brazilcubamexico.dta"

log using "D:\Preg COVID\Log files\TABLE 3 characteristics and outcomes in people with pre-existing conditions.log", replace

tab gender hosp, chi row

tab gender icu, chi row

tab gender death, chi row


tab age10 hosp, chi row

tab age10 icu, chi row

tab age10 death, chi row


tab countrymatch hosp, chi row
```

```
tab countrymatch icu, chi row

tab countrymatch death, chi row


tab year hosp, chi row

tab year icu, chi row

tab year death, chi row


tab diagnosis hosp, chi row

tab diagnosis icu, chi row

tab diagnosis death, chi row


tab cvd hosp, chi row

tab lung hosp, chi row

tab diabetes hosp, chi row

tab kidney hosp, chi row

tab htn hosp, chi row

tab obesity hosp, chi row

tab preg hosp, chi row


tab cvd icu, chi row

tab lung icu, chi row

tab diabetes icu, chi row

tab kidney icu, chi row

tab htn icu, chi row

tab obesity icu, chi row

tab preg icu, exact row
```

```
tab cvd death, chi row

tab lung death, chi row

tab diabetes death, chi row

tab kidney death, chi row

tab htn death, chi row

tab obesity death, chi row

tab preg death, chi row


log close


*RUN RUN

*************table 4 (supp) Association between pre-existing conditions and hosp by age group

log using "xxxx.xxxxx.log", replace

tab hosp cvd, chi col

tab hosp cvd if age10==0, chi col

tab hosp cvd if age10==1, chi col

tab hosp cvd if age10==2, chi col

tab hosp cvd if age10==3, chi col

tab hosp cvd if age10==4, chi col

tab hosp cvd if age10==5, chi col

tab hosp cvd if age10==6, chi col

tab hosp cvd if age10==7, chi col

tab hosp cvd if age10==8, chi col

tab hosp cvd if age10==9, chi col
```

```
tab hosp cvd if age10==10, chi col
tab hosp cvd if age10==11, exact col

tab hosp diabetes , chi col
tab hosp diabetes  if age10==0, chi col
tab hosp diabetes  if age10==1, chi col
tab hosp diabetes  if age10==2, chi col
tab hosp diabetes  if age10==3, chi col
tab hosp diabetes  if age10==4, chi col
tab hosp diabetes  if age10==5, chi col
tab hosp diabetes  if age10==6, chi col
tab hosp diabetes  if age10==7, chi col
tab hosp diabetes  if age10==8, chi col
tab hosp diabetes  if age10==9, chi col
tab hosp diabetes  if age10==10, exact col
tab hosp diabetes  if age10==11, exact col


tab hosp lung, chi col
tab hosp lung if age10==0, chi col
tab hosp lung if age10==1, chi col
tab hosp lung if age10==2, chi col
tab hosp lung if age10==3, chi col
tab hosp lung if age10==4, chi col
tab hosp lung if age10==5, chi col
tab hosp lung if age10==6, chi col
```

```
tab hosp lung if age10==7, chi col
tab hosp lung if age10==8, chi col
tab hosp lung if age10==9, chi col
tab hosp lung if age10==10, exact col
tab hosp lung if age10==11, exact col

tab hosp kidney , chi col
tab hosp kidney  if age10==0, chi col
tab hosp kidney  if age10==1, exact col
tab hosp kidney  if age10==2, chi col
tab hosp kidney  if age10==3, chi col
tab hosp kidney  if age10==4, chi col
tab hosp kidney  if age10==5, chi col
tab hosp kidney  if age10==6, chi col
tab hosp kidney  if age10==7, chi col
tab hosp kidney  if age10==8, chi col
tab hosp kidney  if age10==9, chi col
tab hosp kidney  if age10==10, exact col
tab hosp kidney  if age10==11, exact col

tab hosp htn , chi col
tab hosp htn  if age10==0, chi col
tab hosp htn  if age10==1, exact col
tab hosp htn  if age10==2, chi col
tab hosp htn  if age10==3, chi col
tab hosp htn  if age10==4, chi col
```

```
tab hosp htn  if age10==5, chi col
tab hosp htn  if age10==6, chi col
tab hosp htn  if age10==7, chi col
tab hosp htn  if age10==8, chi col
tab hosp htn  if age10==9, chi col
tab hosp htn  if age10==10, exact col
tab hosp htn  if age10==11, exact col

tab hosp obesity , chi col
tab hosp obesity  if age10==0, chi col
tab hosp obesity  if age10==1, chi col
tab hosp obesity  if age10==2, chi col
tab hosp obesity  if age10==3, chi col
tab hosp obesity  if age10==4, chi col
tab hosp obesity  if age10==5, chi col
tab hosp obesity  if age10==6, chi col
tab hosp obesity  if age10==7, chi col
tab hosp obesity  if age10==8, chi col
tab hosp obesity  if age10==9, chi col
tab hosp obesity  if age10==10, exact col
tab hosp obesity  if age10==11, exact col

tab hosp pregnancy, chi col
tab hosp pregnancy if age10==1, chi col
tab hosp pregnancy if age10==2, chi col
tab hosp pregnancy if age10==3, chi col
```

```
tab hosp pregnancy if age10==4, exact col
log close



*************table 5 (supp) Association between pre-existing conditions and icu by age group
log using "xxx.xxxx.xxx", replace
tab icu cvd , chi col
tab icu cvd  if age10==0, exact col
tab icu cvd  if age10==1, exact col
tab icu cvd  if age10==2, exact col
tab icu cvd  if age10==3, chi col
tab icu cvd  if age10==4, chi col
tab icu cvd  if age10==5, chi col
tab icu cvd  if age10==6, chi col
tab icu cvd  if age10==7, chi col
tab icu cvd  if age10==8, chi col
tab icu cvd  if age10==9, chi col
tab icu cvd  if age10==10, exact col
tab icu cvd  if age10==11, exact col

tab icu diabetes , chi col
tab icu diabetes  if age10==0, exact col
tab icu diabetes  if age10==1, exact col
tab icu diabetes  if age10==2, exact col
tab icu diabetes  if age10==3, chi col
tab icu diabetes  if age10==4, chi col
```

```
tab icu diabetes  if age10==5, chi col
tab icu diabetes  if age10==6, chi col
tab icu diabetes  if age10==7, chi col
tab icu diabetes  if age10==8, chi col
tab icu diabetes  if age10==9, chi col
tab icu diabetes  if age10==10, exact col
tab icu diabetes  if age10==11, exact col


tab icu lung , chi col
tab icu lung  if age10==0, chi col
tab icu lung  if age10==1, exact col
tab icu lung  if age10==2, exact col
tab icu lung  if age10==3, chi col
tab icu lung  if age10==4, chi col
tab icu lung  if age10==5, chi col
tab icu lung  if age10==6, chi col
tab icu lung  if age10==7, chi col
tab icu lung  if age10==8, chi col
tab icu lung  if age10==9, exact col
tab icu lung  if age10==10, exact col
tab icu lung  if age10==11, exact col

tab icu kidney , chi col
tab icu kidney  if age10==0, exact col
tab icu kidney  if age10==1, exact col
```

```
tab icu kidney  if age10==2, exact col

tab icu kidney  if age10==3, exact col

tab icu kidney  if age10==4, exact col

tab icu kidney  if age10==5, chi col

tab icu kidney  if age10==6, chi col

tab icu kidney  if age10==7, chi col

tab icu kidney  if age10==8, chi col

tab icu kidney  if age10==9, exact col

tab icu kidney  if age10==10, exact col

tab icu kidney  if age10==11, exact col


tab icu htn , chi col

tab icu htn  if age10==0, exact col

tab icu htn  if age10==1, exact col

tab icu htn  if age10==2, exact col

tab icu htn  if age10==3, chi col

tab icu htn  if age10==4, chi col

tab icu htn  if age10==5, chi col

tab icu htn  if age10==6, chi col

tab icu htn  if age10==7, chi col

tab icu htn  if age10==8, chi col

tab icu htn  if age10==9, chi col

tab icu htn  if age10==10, exact col

tab icu htn  if age10==11, exact col


tab icu obesity , chi col
```

```
tab icu obesity  if age10==0, exact col

tab icu obesity  if age10==1, exact col

tab icu obesity  if age10==2, chi col

tab icu obesity  if age10==3, chi col

tab icu obesity  if age10==4, chi col

tab icu obesity  if age10==5, chi col

tab icu obesity  if age10==6, chi col

tab icu obesity  if age10==7, chi col

tab icu obesity  if age10==8, chi col

tab icu obesity  if age10==9, exact col

tab icu obesity  if age10==10, exact col

tab icu obesity  if age10==11, exact col


tab icu pregnancy , exact col

tab icu pregnancy  if age10==1, exact col

tab icu pregnancy  if age10==2, exact col

tab icu pregnancy  if age10==3, exact col

tab icu pregnancy  if age10==4, exact col

log close



*************table 6 (supp) Association between pre-existing conditions and mortality by age group

log using "xxx xxxx.log", replace

tab death cvd , chi col

tab death cvd  if age10==0, exact col
```

```
tab death cvd  if age10==1, exact col

tab death cvd  if age10==2, chi col

tab death cvd  if age10==3, chi col

tab death cvd  if age10==4, chi col

tab death cvd  if age10==5, chi col

tab death cvd  if age10==6, chi col

tab death cvd  if age10==7, chi col

tab death cvd  if age10==8, chi col

tab death cvd  if age10==9, chi col

tab death cvd  if age10==10, exact col

tab death cvd  if age10==11, exact col


tab death diabetes , chi col

tab death diabetes  if age10==0, exact col

tab death diabetes  if age10==1, exact col

tab death diabetes  if age10==2, exact col

tab death diabetes  if age10==3, chi col

tab death diabetes  if age10==4, chi col

tab death diabetes  if age10==5, chi col

tab death diabetes  if age10==6, chi col

tab death diabetes  if age10==7, chi col

tab death diabetes  if age10==8, chi col

tab death diabetes  if age10==9, chi col

tab death diabetes  if age10==10, exact col

tab death diabetes  if age10==11, exact col
```

```
tab death lung , chi col

tab death lung  if age10==0, exact col

tab death lung  if age10==1, exact col

tab death lung  if age10==2, chi col

tab death lung  if age10==3, chi col

tab death lung  if age10==4, chi col

tab death lung  if age10==5, chi col

tab death lung  if age10==6, chi col

tab death lung  if age10==7, chi col

tab death lung  if age10==8, chi col

tab death lung  if age10==9, chi col

tab death lung  if age10==10, exact col

tab death lung  if age10==11, exact col


tab death kidney , chi col

tab death kidney  if age10==0, exact col

tab death kidney  if age10==1, exact col

tab death kidney  if age10==2, exact col

tab death kidney  if age10==3, exact col

tab death kidney  if age10==4, chi col

tab death kidney  if age10==5, chi col

tab death kidney  if age10==6, chi col

tab death kidney  if age10==7, chi col

tab death kidney  if age10==8, chi col

tab death kidney  if age10==9, chi col
```

```
tab death kidney  if age10==10, exact col
tab death kidney  if age10==11, exact col

tab death htn , chi col
tab death htn  if age10==0, exact col
tab death htn  if age10==1, exact col
tab death htn  if age10==2, exact col
tab death htn  if age10==3, chi col
tab death htn  if age10==4, chi col
tab death htn  if age10==5, chi col
tab death htn  if age10==6, chi col
tab death htn  if age10==7, chi col
tab death htn  if age10==8, chi col
tab death htn  if age10==9, chi col
tab death htn  if age10==10, exact col
tab death htn  if age10==11, exact col

tab death obesity , chi col
tab death obesity  if age10==0, exact col
tab death obesity  if age10==1, exact col
tab death obesity  if age10==2, chi col
tab death obesity  if age10==3, chi col
tab death obesity  if age10==4, chi col
tab death obesity  if age10==5, chi col
tab death obesity  if age10==6, chi col
tab death obesity  if age10==7, chi col
```

```
tab death obesity  if age10==8, chi col

tab death obesity  if age10==9, chi col

tab death obesity  if age10==10, exact col

tab death obesity  if age10==11, exact col


tab death pregnancy , chi col

tab death pregnancy  if age10==1, exact col

tab death pregnancy  if age10==2, exact col

tab death pregnancy  if age10==3, exact col

tab death pregnancy  if age10==4, exact col

log close
```

```
**************table5--Association between cardiovascular diseases and severity outcome
```

```
log using "D:\Preg COVID\Log files\table5--Association between cardiovascular diseases and severity outcome.log", replace

drop if age==12|age==13|age==15

*total row 1

logistic hosp cvd

logistic hosp cvd i.gender i.countrymatch  i.diabetes i.obesity i.age10

logistic icu cvd

logistic icu cvd i.gender i.countrymatch  i.diabetes i.obesity i.age10

logistic death cvd

logistic death cvd i.gender i.countrymatch  i.diabetes i.obesity i.age10

*age group (0-9)

logistic hosp cvd if age10==0

logistic hosp cvd i.gender i.countrymatch  i.diabetes i.obesity if age10==0

logistic icu cvd if age10==0

logistic icu cvd i.gender i.countrymatch  i.diabetes i.obesity if age10==0

logistic death cvd if age10==0

logistic death cvd i.gender i.countrymatch  i.diabetes i.obesity if age10==0

*age group (10-19)

logistic hosp cvd if age10==1

logistic hosp cvd i.gender i.countrymatch  i.diabetes i.obesity if age10==1

logistic icu cvd if age10==1

logistic icu cvd i.gender i.countrymatch  i.diabetes i.obesity if age10==1

logistic death cvd if age10==1

logistic death cvd i.gender i.countrymatch  i.diabetes i.obesity if age10==1

*age group (20-29)

logistic hosp cvd if age10==2
```

```
logistic hosp cvd i.gender i.countrymatch  i.diabetes i.obesity if age10==2

logistic icu cvd if age10==2

logistic icu cvd i.gender i.countrymatch  i.diabetes i.obesity if age10==2

logistic death cvd if age10==2

logistic death cvd i.gender i.countrymatch  i.diabetes i.obesity if age10==2

*age group (30-39)

logistic hosp cvd if age10==3

logistic hosp cvd i.gender i.countrymatch  i.diabetes i.obesity if age10==3

logistic icu cvd if age10==3

logistic icu cvd i.gender i.countrymatch  i.diabetes i.obesity if age10==3

logistic death cvd if age10==3

logistic death cvd i.gender i.countrymatch  i.diabetes i.obesity if age10==3

*age group (40-49)

logistic hosp cvd if age10==4

logistic hosp cvd i.gender i.countrymatch  i.diabetes i.obesity if age10==4

logistic icu cvd if age10==4

logistic icu cvd i.gender i.countrymatch  i.diabetes i.obesity if age10==4

logistic death cvd if age10==4

logistic death cvd i.gender i.countrymatch  i.diabetes i.obesity if age10==4

*age group (50-59)

logistic hosp cvd if age10==5

logistic hosp cvd i.gender i.countrymatch  i.diabetes i.obesity if age10==5

logistic icu cvd if age10==5

logistic icu cvd i.gender i.countrymatch  i.diabetes i.obesity if age10==5

logistic death cvd if age10==5

logistic death cvd i.gender i.countrymatch  i.diabetes i.obesity if age10==5
```

```
*age group (60-69)
logistic hosp cvd if age10==6
logistic hosp cvd i.gender i.countrymatch  i.diabetes i.obesity if age10==6
logistic icu cvd if age10==6
logistic icu cvd i.gender i.countrymatch  i.diabetes i.obesity if age10==6
logistic death cvd if age10==6
logistic death cvd i.gender i.countrymatch  i.diabetes i.obesity if age10==6
*age group (70-79)
logistic hosp cvd if age10==7
logistic hosp cvd i.gender i.countrymatch  i.diabetes i.obesity if age10==7
logistic icu cvd if age10==7
logistic icu cvd i.gender i.countrymatch  i.diabetes i.obesity if age10==7
logistic death cvd if age10==7
logistic death cvd i.gender i.countrymatch  i.diabetes i.obesity if age10==7
*age group (80-89)
logistic hosp cvd if age10==8
logistic hosp cvd i.gender i.countrymatch  i.diabetes i.obesity if age10==8
logistic icu cvd if age10==8
logistic icu cvd i.gender i.countrymatch  i.diabetes i.obesity if age10==8
logistic death cvd if age10==8
logistic death cvd i.gender i.countrymatch  i.diabetes i.obesity if age10==8
*age group (90-99)
logistic hosp cvd if age10==9
logistic hosp cvd i.gender i.countrymatch  i.diabetes i.obesity if age10==9
logistic icu cvd if age10==9
logistic icu cvd i.gender i.countrymatch  i.diabetes i.obesity if age10==9
```

logistic death cvd if age10==9

logistic death cvd i.gender i.countrymatch i.diabetes i.obesity if age10==9

*age group (100-109)

logistic hosp cvd if age10==10

logistic hosp cvd i.gender i.countrymatch i.diabetes i.obesity if age10==10

logistic icu cvd if age10==10

logistic icu cvd i.gender i.countrymatch i.diabetes i.obesity if age10==10

logistic death cvd if age10==10

logistic death cvd i.gender i.countrymatch i.diabetes i.obesity if age10==10

*age group (110-119)

logistic hosp cvd if age10==11

logistic hosp cvd i.gender i.countrymatch i.diabetes i.obesity if age10==11

logistic icu cvd if age10==11

logistic icu cvd i.gender i.countrymatch i.diabetes i.obesity if age10==11

logistic death cvd if age10==11

logistic death cvd i.gender i.countrymatch i.diabetes i.obesity if age10==11

log close

******************table6--Association between lung diseases and severity outcome

log using "D:\Preg COVID\Log files\table6--Association between lung diseases and severity outcome.log", replace

*total row 1

logistic hosp lung

logistic hosp lung i.gender i.countrymatch   i.obesity i.age10

logistic icu lung

logistic icu lung i.gender i.countrymatch   i.obesity i.age10

```
logistic death lung

logistic death lung i.gender i.countrymatch   i.obesity i.age10

*age group (0-9)

logistic hosp lung if age10==0

logistic hosp lung i.gender i.countrymatch   i.obesity if age10==0

logistic icu lung if age10==0

logistic icu lung i.gender i.countrymatch   i.obesity if age10==0

logistic death lung if age10==0

logistic death lung i.gender i.countrymatch   i.obesity if age10==0

*age group (10-19)

logistic hosp lung if age10==1

logistic hosp lung i.gender i.countrymatch   i.obesity if age10==1

logistic icu lung if age10==1

logistic icu lung i.gender i.countrymatch   i.obesity if age10==1

logistic death lung if age10==1

logistic death lung i.gender i.countrymatch   i.obesity if age10==1

*age group (20-29)

logistic hosp lung if age10==2

logistic hosp lung i.gender i.countrymatch   i.obesity if age10==2

logistic icu lung if age10==2

logistic icu lung i.gender i.countrymatch   i.obesity if age10==2

logistic death lung if age10==2

logistic death lung i.gender i.countrymatch   i.obesity if age10==2

*age group (30-39)

logistic hosp lung if age10==3

logistic hosp lung i.gender i.countrymatch   i.obesity if age10==3
```

logistic icu lung if age10==3

logistic icu lung i.gender i.countrymatch   i.obesity if age10==3

logistic death lung if age10==3

logistic death lung i.gender i.countrymatch   i.obesity if age10==3

*age group (40-49)

logistic hosp lung if age10==4

logistic hosp lung i.gender i.countrymatch   i.obesity if age10==4

logistic icu lung if age10==4

logistic icu lung i.gender i.countrymatch   i.obesity if age10==4

logistic death lung if age10==4

logistic death lung i.gender i.countrymatch   i.obesity if age10==4

*age group (50-59)

logistic hosp lung if age10==5

logistic hosp lung i.gender i.countrymatch   i.obesity if age10==5

logistic icu lung if age10==5

logistic icu lung i.gender i.countrymatch   i.obesity if age10==5

logistic death lung if age10==5

logistic death lung i.gender i.countrymatch   i.obesity if age10==5

*age group (60-69)

logistic hosp lung if age10==6

logistic hosp lung i.gender i.countrymatch   i.obesity if age10==6

logistic icu lung if age10==6

logistic icu lung i.gender i.countrymatch   i.obesity if age10==6

logistic death lung if age10==6

logistic death lung i.gender i.countrymatch   i.obesity if age10==6

*age group (70-79)

```
logistic hosp lung if age10==7
logistic hosp lung i.gender i.countrymatch   i.obesity if age10==7
logistic icu lung if age10==7
logistic icu lung i.gender i.countrymatch   i.obesity if age10==7
logistic death lung if age10==7
logistic death lung i.gender i.countrymatch   i.obesity if age10==7
*age group (80-89)
logistic hosp lung if age10==8
logistic hosp lung i.gender i.countrymatch   i.obesity if age10==8
logistic icu lung if age10==8
logistic icu lung i.gender i.countrymatch   i.obesity if age10==8
logistic death lung if age10==8
logistic death lung i.gender i.countrymatch   i.obesity if age10==8
*age group (90-99)
logistic hosp lung if age10==9
logistic hosp lung i.gender i.countrymatch   i.obesity if age10==9
logistic icu lung if age10==9
logistic icu lung i.gender i.countrymatch   i.obesity if age10==9
logistic death lung if age10==9
logistic death lung i.gender i.countrymatch   i.obesity if age10==9
*age group (100-109)
logistic hosp lung if age10==10
logistic hosp lung i.gender i.countrymatch   i.obesity if age10==10
logistic icu lung if age10==10
logistic icu lung i.gender i.countrymatch   i.obesity if age10==10
logistic death lung if age10==10
```

logistic death lung i.gender i.countrymatch   i.obesity if age10==10

*age group (110-119)

logistic hosp lung if age10==11

logistic hosp lung i.gender i.countrymatch   i.obesity if age10==11

logistic icu lung if age10==11

logistic icu lung i.gender i.countrymatch   i.obesity if age10==11

logistic death lung if age10==11

logistic death lung i.gender i.countrymatch   i.obesity if age10==11

log close

***************************table7--Association between diabetes and severity outcome

log using "D:\Preg COVID\Log files\table7--Association between diabetes and severity outcome.log", replace

*total row 1

logistic hosp diabetes

logistic hosp diabetes i.gender i.countrymatch i.cvd i.obesity i.age10

logistic icu diabetes

logistic icu diabetes i.gender i.countrymatch i.cvd i.obesity i.age10

logistic death diabetes

logistic death diabetes i.gender i.countrymatch i.cvd i.obesity i.age10

*age group (0-9)

logistic hosp diabetes if age10==0

logistic hosp diabetes i.gender i.countrymatch i.cvd i.obesity if age10==0

logistic icu diabetes if age10==0

logistic icu diabetes i.gender i.countrymatch i.cvd i.obesity if age10==0

```
logistic death diabetes if age10==0

logistic death diabetes i.gender i.countrymatch  i.cvd i.obesity if age10==0

*age group (10-19)

logistic hosp diabetes if age10==1

logistic hosp diabetes i.gender i.countrymatch  i.cvd i.obesity if age10==1

logistic icu diabetes if age10==1

logistic icu diabetes i.gender i.countrymatch  i.cvd i.obesity if age10==1

logistic death diabetes if age10==1

logistic death diabetes i.gender i.countrymatch  i.cvd i.obesity if age10==1

*age group (20-29)

logistic hosp diabetes if age10==2

logistic hosp diabetes i.gender i.countrymatch  i.cvd i.obesity if age10==2

logistic icu diabetes if age10==2

logistic icu diabetes i.gender i.countrymatch  i.cvd i.obesity if age10==2

logistic death diabetes if age10==2

logistic death diabetes i.gender i.countrymatch  i.cvd i.obesity if age10==2

*age group (30-39)

logistic hosp diabetes if age10==3

logistic hosp diabetes i.gender i.countrymatch  i.cvd i.obesity if age10==3

logistic icu diabetes if age10==3

logistic icu diabetes i.gender i.countrymatch  i.cvd i.obesity if age10==3

logistic death diabetes if age10==3

logistic death diabetes i.gender i.countrymatch  i.cvd i.obesity if age10==3

*age group (40-49)

logistic hosp diabetes if age10==4

logistic hosp diabetes i.gender i.countrymatch  i.cvd i.obesity if age10==4
```

```
logistic icu diabetes if age10==4

logistic icu diabetes i.gender i.countrymatch  i.cvd i.obesity if age10==4

logistic death diabetes if age10==4

logistic death diabetes i.gender i.countrymatch  i.cvd i.obesity if age10==4

*age group (50-59)

logistic hosp diabetes if age10==5

logistic hosp diabetes i.gender i.countrymatch  i.cvd i.obesity if age10==5

logistic icu diabetes if age10==5

logistic icu diabetes i.gender i.countrymatch  i.cvd i.obesity if age10==5

logistic death diabetes if age10==5

logistic death diabetes i.gender i.countrymatch  i.cvd i.obesity if age10==5

*age group (60-69)

logistic hosp diabetes if age10==6

logistic hosp diabetes i.gender i.countrymatch  i.cvd i.obesity if age10==6

logistic icu diabetes if age10==6

logistic icu diabetes i.gender i.countrymatch  i.cvd i.obesity if age10==6

logistic death diabetes if age10==6

logistic death diabetes i.gender i.countrymatch  i.cvd i.obesity if age10==6

*age group (70-79)

logistic hosp diabetes if age10==7

logistic hosp diabetes i.gender i.countrymatch  i.cvd i.obesity if age10==7

logistic icu diabetes if age10==7

logistic icu diabetes i.gender i.countrymatch  i.cvd i.obesity if age10==7

logistic death diabetes if age10==7

logistic death diabetes i.gender i.countrymatch  i.cvd i.obesity if age10==7

*age group (80-89)
```

```
logistic hosp diabetes if age10==8

logistic hosp diabetes i.gender i.countrymatch  i.cvd i.obesity if age10==8

logistic icu diabetes if age10==8

logistic icu diabetes i.gender i.countrymatch  i.cvd i.obesity if age10==8

logistic death diabetes if age10==8

logistic death diabetes i.gender i.countrymatch  i.cvd i.obesity if age10==8

*age group (90-99)

logistic hosp diabetes if age10==9

logistic hosp diabetes i.gender i.countrymatch  i.cvd i.obesity if age10==9

logistic icu diabetes if age10==9

logistic icu diabetes i.gender i.countrymatch  i.cvd i.obesity if age10==9

logistic death diabetes if age10==9

logistic death diabetes i.gender i.countrymatch  i.cvd i.obesity if age10==9

*age group (100-109)

logistic hosp diabetes if age10==10

logistic hosp diabetes i.gender i.countrymatch  i.cvd i.obesity if age10==10

logistic icu diabetes if age10==10

logistic icu diabetes i.gender i.countrymatch  i.cvd i.obesity if age10==10

logistic death diabetes if age10==10

logistic death diabetes i.gender i.countrymatch  i.cvd i.obesity if age10==10

*age group (110-119)

logistic hosp diabetes if age10==11

logistic hosp diabetes i.gender i.countrymatch  i.cvd i.obesity if age10==11

logistic icu diabetes if age10==11

logistic icu diabetes i.gender i.countrymatch  i.cvd i.obesity if age10==11

logistic death diabetes if age10==11
```

```
logistic death diabetes i.gender i.countrymatch  i.cvd i.obesity if age10==11

log close


*************table8--Association between kidney diseases and severity outcome

log using "D:\Preg COVID\Log files\table8--Association between kidney diseases and severity outcome.log", replace


*total row 1

logistic hosp kidney

logistic hosp kidney i.gender i.countrymatch  i.cvd i.diabetes i.obesity i.age10

logistic icu kidney

logistic icu kidney i.gender i.countrymatch  i.cvd i.diabetes i.obesity i.age10

logistic death kidney

logistic death kidney i.gender i.countrymatch  i.cvd i.diabetes i.obesity i.age10

*age group (0-9)

logistic hosp kidney if age10==0

logistic hosp kidney i.gender i.countrymatch  i.cvd i.diabetes i.obesity if age10==0

logistic icu kidney if age10==0

logistic icu kidney i.gender i.countrymatch  i.cvd i.diabetes i.obesity if age10==0

logistic death kidney if age10==0

logistic death kidney i.gender i.countrymatch  i.cvd i.diabetes i.obesity if age10==0

*age group (10-19)

logistic hosp kidney if age10==1

logistic hosp kidney i.gender i.countrymatch  i.cvd i.diabetes i.obesity if age10==1

logistic icu kidney if age10==1

logistic icu kidney i.gender i.countrymatch  i.cvd i.diabetes i.obesity if age10==1
```

```
logistic death kidney if age10==1

logistic death kidney i.gender i.countrymatch  i.cvd i.diabetes i.obesity if age10==1

*age group (20-29)

logistic hosp kidney if age10==2

logistic hosp kidney i.gender i.countrymatch  i.cvd i.diabetes i.obesity if age10==2

logistic icu kidney if age10==2

logistic icu kidney i.gender i.countrymatch  i.cvd i.diabetes i.obesity if age10==2

logistic death kidney if age10==2

logistic death kidney i.gender i.countrymatch  i.cvd i.diabetes i.obesity if age10==2

*age group (30-39)

logistic hosp kidney if age10==3

logistic hosp kidney i.gender i.countrymatch  i.cvd i.diabetes i.obesity if age10==3

logistic icu kidney if age10==3

logistic icu kidney i.gender i.countrymatch  i.cvd i.diabetes i.obesity if age10==3

logistic death kidney if age10==3

logistic death kidney i.gender i.countrymatch  i.cvd i.diabetes i.obesity if age10==3

*age group (40-49)

logistic hosp kidney if age10==4

logistic hosp kidney i.gender i.countrymatch  i.cvd i.diabetes i.obesity if age10==4

logistic icu kidney if age10==4

logistic icu kidney i.gender i.countrymatch  i.cvd i.diabetes i.obesity if age10==4

logistic death kidney if age10==4

logistic death kidney i.gender i.countrymatch  i.cvd i.diabetes i.obesity if age10==4

*age group (50-59)

logistic hosp kidney if age10==5

logistic hosp kidney i.gender i.countrymatch  i.cvd i.diabetes i.obesity if age10==5
```

logistic icu kidney if age10==5

logistic icu kidney i.gender i.countrymatch i.cvd i.diabetes i.obesity if age10==5

logistic death kidney if age10==5

logistic death kidney i.gender i.countrymatch i.cvd i.diabetes i.obesity if age10==5

*age group (60-69)

logistic hosp kidney if age10==6

logistic hosp kidney i.gender i.countrymatch i.cvd i.diabetes i.obesity if age10==6

logistic icu kidney if age10==6

logistic icu kidney i.gender i.countrymatch i.cvd i.diabetes i.obesity if age10==6

logistic death kidney if age10==6

logistic death kidney i.gender i.countrymatch i.cvd i.diabetes i.obesity if age10==6

*age group (70-79)

logistic hosp kidney if age10==7

logistic hosp kidney i.gender i.countrymatch i.cvd i.diabetes i.obesity if age10==7

logistic icu kidney if age10==7

logistic icu kidney i.gender i.countrymatch i.cvd i.diabetes i.obesity if age10==7

logistic death kidney if age10==7

logistic death kidney i.gender i.countrymatch i.cvd i.diabetes i.obesity if age10==7

*age group (80-89)

logistic hosp kidney if age10==8

logistic hosp kidney i.gender i.countrymatch i.cvd i.diabetes i.obesity if age10==8

logistic icu kidney if age10==8

logistic icu kidney i.gender i.countrymatch i.cvd i.diabetes i.obesity if age10==8

logistic death kidney if age10==8

logistic death kidney i.gender i.countrymatch i.cvd i.diabetes i.obesity if age10==8

*age group (90-99)

```
logistic hosp kidney if age10==9

logistic hosp kidney i.gender i.countrymatch  i.cvd i.diabetes i.obesity if age10==9

logistic icu kidney if age10==9

logistic icu kidney i.gender i.countrymatch  i.cvd i.diabetes i.obesity if age10==9

logistic death kidney if age10==9

logistic death kidney i.gender i.countrymatch  i.cvd i.diabetes i.obesity if age10==9

*age group (100-109)

logistic hosp kidney if age10==10

logistic hosp kidney i.gender i.countrymatch  i.cvd i.diabetes i.obesity if age10==10

logistic icu kidney if age10==10

logistic icu kidney i.gender i.countrymatch  i.cvd i.diabetes i.obesity if age10==10

logistic death kidney if age10==10

logistic death kidney i.gender i.countrymatch  i.cvd i.diabetes i.obesity if age10==10

*age group (110-119)

logistic hosp kidney if age10==11

logistic hosp kidney i.gender i.countrymatch  i.cvd i.diabetes i.obesity if age10==11

logistic icu kidney if age10==11

logistic icu kidney i.gender i.countrymatch  i.cvd i.diabetes i.obesity if age10==11

logistic death kidney if age10==11

logistic death kidney i.gender i.countrymatch  i.cvd i.diabetes i.obesity if age10==11

log close


***********table9--Association between obesity and severity outcome

log using "D:\Preg COVID\Log files\table9--Association between obesity and severity
outcome.log", replace
```

```
*total row 1
logistic hosp obesity
logistic hosp obesity i.gender i.countrymatch  i.cvd i.diabetes i.age10
logistic icu obesity
logistic icu obesity i.gender i.countrymatch  i.cvd i.diabetes  i.age10
logistic death obesity
logistic death obesity i.gender i.countrymatch  i.cvd i.diabetes  i.age10
*age group (0-9)
logistic hosp obesity if age10==0
logistic hosp obesity i.gender i.countrymatch  i.cvd i.diabetes   if age10==0
logistic icu obesity if age10==0
logistic icu obesity i.gender i.countrymatch  i.cvd i.diabetes   if age10==0
logistic death obesity if age10==0
logistic death obesity i.gender i.countrymatch  i.cvd i.diabetes   if age10==0
*age group (10-19)
logistic hosp obesity if age10==1
logistic hosp obesity i.gender i.countrymatch  i.cvd i.diabetes   if age10==1
logistic icu obesity if age10==1
logistic icu obesity i.gender i.countrymatch  i.cvd i.diabetes   if age10==1
logistic death obesity if age10==1
logistic death obesity i.gender i.countrymatch  i.cvd i.diabetes   if age10==1
*age group (20-29)
logistic hosp obesity if age10==2
logistic hosp obesity i.gender i.countrymatch  i.cvd i.diabetes   if age10==2
logistic icu obesity if age10==2
logistic icu obesity i.gender i.countrymatch  i.cvd i.diabetes   if age10==2
```

```
logistic death obesity if age10==2

logistic death obesity i.gender i.countrymatch  i.cvd i.diabetes   if age10==2

*age group (30-39)

logistic hosp obesity if age10==3

logistic hosp obesity i.gender i.countrymatch  i.cvd i.diabetes   if age10==3

logistic icu obesity if age10==3

logistic icu obesity i.gender i.countrymatch  i.cvd i.diabetes   if age10==3

logistic death obesity if age10==3

logistic death obesity i.gender i.countrymatch  i.cvd i.diabetes   if age10==3

*age group (40-49)

logistic hosp obesity if age10==4

logistic hosp obesity i.gender i.countrymatch  i.cvd i.diabetes   if age10==4

logistic icu obesity if age10==4

logistic icu obesity i.gender i.countrymatch  i.cvd i.diabetes   if age10==4

logistic death obesity if age10==4

logistic death obesity i.gender i.countrymatch  i.cvd i.diabetes   if age10==4

*age group (50-59)

logistic hosp obesity if age10==5

logistic hosp obesity i.gender i.countrymatch  i.cvd i.diabetes   if age10==5

logistic icu obesity if age10==5

logistic icu obesity i.gender i.countrymatch  i.cvd i.diabetes   if age10==5

logistic death obesity if age10==5

logistic death obesity i.gender i.countrymatch  i.cvd i.diabetes   if age10==5

*age group (60-69)

logistic hosp obesity if age10==6

logistic hosp obesity i.gender i.countrymatch  i.cvd i.diabetes   if age10==6
```

```
logistic icu obesity if age10==6

logistic icu obesity i.gender i.countrymatch  i.cvd i.diabetes   if age10==6

logistic death obesity if age10==6

logistic death obesity i.gender i.countrymatch  i.cvd i.diabetes   if age10==6

*age group (70-79)

logistic hosp obesity if age10==7

logistic hosp obesity i.gender i.countrymatch  i.cvd i.diabetes   if age10==7

logistic icu obesity if age10==7

logistic icu obesity i.gender i.countrymatch  i.cvd i.diabetes   if age10==7

logistic death obesity if age10==7

logistic death obesity i.gender i.countrymatch  i.cvd i.diabetes   if age10==7

*age group (80-89)

logistic hosp obesity if age10==8

logistic hosp obesity i.gender i.countrymatch  i.cvd i.diabetes   if age10==8

logistic icu obesity if age10==8

logistic icu obesity i.gender i.countrymatch  i.cvd i.diabetes   if age10==8

logistic death obesity if age10==8

logistic death obesity i.gender i.countrymatch  i.cvd i.diabetes   if age10==8

*age group (90-99)

logistic hosp obesity if age10==9

logistic hosp obesity i.gender i.countrymatch  i.cvd i.diabetes   if age10==9

logistic icu obesity if age10==9

logistic icu obesity i.gender i.countrymatch  i.cvd i.diabetes   if age10==9

logistic death obesity if age10==9

logistic death obesity i.gender i.countrymatch  i.cvd i.diabetes   if age10==9

*age group (100-109)
```

logistic hosp obesity if age10==10

logistic hosp obesity i.gender i.countrymatch i.cvd i.diabetes   if age10==10

logistic icu obesity if age10==10

logistic icu obesity i.gender i.countrymatch i.cvd i.diabetes   if age10==10

logistic death obesity if age10==10

logistic death obesity i.gender i.countrymatch i.cvd i.diabetes   if age10==10

*age group (110-119)

logistic hosp obesity if age10==11

logistic hosp obesity i.gender i.countrymatch i.cvd i.diabetes   if age10==11

logistic icu obesity if age10==11

logistic icu obesity i.gender i.countrymatch i.cvd i.diabetes   if age10==11

logistic death obesity if age10==11

logistic death obesity i.gender i.countrymatch i.cvd i.diabetes   if age10==11

log close

**************table10--Association between hypertension and severity outcome

log using "D:\Preg COVID\Log files\table10--Association between htn and severity outcome.log", replace

*total row 1

logistic hosp htn

logistic hosp htn i.gender i.countrymatch i.diabetes i.obesity i.age10

logistic icu htn

logistic icu htn i.gender i.countrymatch i.diabetes i.obesity i.age10

logistic death htn

```
logistic death htn i.gender i.countrymatch  i.diabetes i.obesity i.age10

*age group (0-9)

logistic hosp htn if age10==0

logistic hosp htn i.gender i.countrymatch  i.diabetes i.obesity if age10==0

logistic icu htn if age10==0

logistic icu htn i.gender i.countrymatch  i.diabetes i.obesity if age10==0

logistic death htn if age10==0

logistic death htn i.gender i.countrymatch  i.diabetes i.obesity if age10==0

*age group (10-19)

logistic hosp htn if age10==1

logistic hosp htn i.gender i.countrymatch  i.diabetes i.obesity if age10==1

logistic icu htn if age10==1

logistic icu htn i.gender i.countrymatch  i.diabetes i.obesity if age10==1

logistic death htn if age10==1

logistic death htn i.gender i.countrymatch  i.diabetes i.obesity if age10==1

*age group (20-29)

logistic hosp htn if age10==2

logistic hosp htn i.gender i.countrymatch  i.diabetes i.obesity if age10==2

logistic icu htn if age10==2

logistic icu htn i.gender i.countrymatch  i.diabetes i.obesity if age10==2

logistic death htn if age10==2

logistic death htn i.gender i.countrymatch  i.diabetes i.obesity if age10==2

*age group (30-39)

logistic hosp htn if age10==3

logistic hosp htn i.gender i.countrymatch  i.diabetes i.obesity if age10==3

logistic icu htn if age10==3
```

```
logistic icu htn i.gender i.countrymatch  i.diabetes i.obesity if age10==3

logistic death htn if age10==3

logistic death htn i.gender i.countrymatch  i.diabetes i.obesity if age10==3

*age group (40-49)

logistic hosp htn if age10==4

logistic hosp htn i.gender i.countrymatch  i.diabetes i.obesity if age10==4

logistic icu htn if age10==4

logistic icu htn i.gender i.countrymatch  i.diabetes i.obesity if age10==4

logistic death htn if age10==4

logistic death htn i.gender i.countrymatch  i.diabetes i.obesity if age10==4

*age group (50-59)

logistic hosp htn if age10==5

logistic hosp htn i.gender i.countrymatch  i.diabetes i.obesity if age10==5

logistic icu htn if age10==5

logistic icu htn i.gender i.countrymatch  i.diabetes i.obesity if age10==5

logistic death htn if age10==5

logistic death htn i.gender i.countrymatch  i.diabetes i.obesity if age10==5

*age group (60-69)

logistic hosp htn if age10==6

logistic hosp htn i.gender i.countrymatch  i.diabetes i.obesity if age10==6

logistic icu htn if age10==6

logistic icu htn i.gender i.countrymatch  i.diabetes i.obesity if age10==6

logistic death htn if age10==6

logistic death htn i.gender i.countrymatch  i.diabetes i.obesity if age10==6

*age group (70-79)

logistic hosp htn if age10==7
```

```
logistic hosp htn i.gender i.countrymatch  i.diabetes i.obesity if age10==7

logistic icu htn if age10==7

logistic icu htn i.gender i.countrymatch  i.diabetes i.obesity if age10==7

logistic death htn if age10==7

logistic death htn i.gender i.countrymatch  i.diabetes i.obesity if age10==7

*age group (80-89)

logistic hosp htn if age10==8

logistic hosp htn i.gender i.countrymatch  i.diabetes i.obesity if age10==8

logistic icu htn if age10==8

logistic icu htn i.gender i.countrymatch  i.diabetes i.obesity if age10==8

logistic death htn if age10==8

logistic death htn i.gender i.countrymatch  i.diabetes i.obesity if age10==8

*age group (90-99)

logistic hosp htn if age10==9

logistic hosp htn i.gender i.countrymatch  i.diabetes i.obesity if age10==9

logistic icu htn if age10==9

logistic icu htn i.gender i.countrymatch  i.diabetes i.obesity if age10==9

logistic death htn if age10==9

logistic death htn i.gender i.countrymatch  i.diabetes i.obesity if age10==9

*age group (100-109)

logistic hosp htn if age10==10

logistic hosp htn i.gender i.countrymatch  i.diabetes i.obesity if age10==10

logistic icu htn if age10==10

logistic icu htn i.gender i.countrymatch  i.diabetes i.obesity if age10==10

logistic death htn if age10==10

logistic death htn i.gender i.countrymatch  i.diabetes i.obesity if age10==10
```

*age group (110-119)

logistic hosp htn if age10==11

logistic hosp htn i.gender i.countrymatch i.diabetes i.obesity if age10==11

logistic icu htn if age10==11

logistic icu htn i.gender i.countrymatch i.diabetes i.obesity if age10==11

logistic death htn if age10==11

logistic death htn i.gender i.countrymatch i.diabetes i.obesity if age10==11

log close

**************table11--Association between pregnancy and severity outcome

log using "D:\Preg COVID\Log files\table11--Association between pregnancy and severity outcome.log", replace

keep if gender==1

keep if age10==1 |age10==2 |age10==3 |age10==4

keep if countrymatch==19

*total row 1

logistic hosp pregnancy

logistic hosp pregnancy i.age10

logistic icu pregnancy

logistic icu pregnancy i.age10

logistic death pregnancy

logistic death pregnancy i.age10

*age group (10-19)

logistic hosp pregnancy if age10==1

logistic icu pregnancy if age10==1

```
logistic death pregnancy if age10==1

*age group (20-29)

logistic hosp pregnancy if age10==2

logistic icu pregnancy if age10==2

logistic death pregnancy if age10==2

*age group (30-39)

logistic hosp pregnancy if age10==3

logistic icu pregnancy if age10==3

logistic death pregnancy if age10==3

*age group (40-49)

logistic hosp pregnancy if age10==4

logistic icu pregnancy if age10==4

logistic death pregnancy if age10==4


log close
```
